# Intraspecific Karyotypic Differentiation in the *Lycoris aurea* Complex Revealed by Fluorochrome Banding and rDNA Fluorescence In Situ Hybridization

**DOI:** 10.3390/plants15192993

**Published:** 2026-09-30

**Authors:** Chaowen She, Xianghui Jiang, Miaohua Quan, Weizheng Kong, Yuqian Tang, Yiqing Yang, Junyue Wu, Zhuohui Zhu, Jie Zhou

**Affiliations:** 1 Key Laboratory of Research and Utilization of Ethnomedicinal Plant Resources of Hunan Province, Huaihua University, Huaihua 418008, China; jxh@hhtc.edu.cn (X.J.); hhqmh100@163.com (M.Q.); 2 College of Biological and Food Engineering, Huaihua University, Huaihua 418008, China; kongweizheng_2026@163.com (W.K.); 15874831726@163.com (Y.T.); yyq970504@163.com (Y.Y.); zgcs110@163.com (J.W.); 18855279684@163.com (Z.Z.); as_chow@163.com (J.Z.)

**Keywords:** *Lycoris aurea*, intraspecific variation, karyotype, dysploidy, ribosomal DNA (rDNA), fluorochrome banding, fluorescence in situ hybridization (FISH), cytogeography

## Abstract

The *Lycoris aurea* (L’Hér.) Herb. complex—valued for its medicinal properties and horticultural applications—is characterized by extensive intraspecific dysploidy. However, a comprehensive molecular cytogenetic framework elucidating intraspecific karyotypic differentiation and the underlying evolutionary mechanisms has yet to be established. By integrating fluorochrome banding with 5S and 45S rDNA fluorescence in situ hybridization (FISH), we performed a comparative molecular cytogenetic karyotyping of 20 natural populations and synthesized these results with previously published cytogenetic data to conduct a rigorous cytogeographic analysis of this species complex. Three distinct cytotypes were identified: cytotype A (2*n* = 14), cytotype B (2*n* = 15), and cytotype C (2*n* = 16). Both multivariate karyomorphometric analyses and comparison of DAPI banding and rDNA FISH patterns revealed high inter-population karyotypic uniformity in cytotype A, yet pronounced intra-cytotypic karyotypic polymorphism in cytotype C. Integrated cytogenetic evidence supports a working hypothesis wherein cytotype C likely represents the ancestral karyotype, while cytotypes A and B appear to have originated from cytotype C through two and one Robertsonian fusion events, respectively—thereby implying two independent, parallel evolutionary pathways: C → A and C → B. The evolutionary biogeographic framework for this species complex—derived from hypothesis-generating integrative inference—proposes that Quaternary climate oscillations, topographic barriers, and ecological niche divergence may have acted synergistically to shape the current parapatric distribution pattern of the three cytotypes.

## 1. Introduction

*Lycoris aurea* (L’Hér.) Herb. is a perennial herbaceous plant belonging to the family Amaryllidaceae and the genus *Lycoris*. It is commonly referred to as the “Golden Spider Lily”, owing to its striking inflorescences characterized by vibrant golden-yellow tepals. *L. aurea* is native to China, Japan, India, Indonesia, Laos, Myanmar, Pakistan, Thailand and Vietnam [1]. In China, it is distributed in Fujian, Gansu, Guangdong, Guangxi, Guizhou, Henan, Hubei, Hunan, Jiangsu, Jiangxi, Shaanxi, Sichuan, Chongqing, Taiwan, Yunnan and Zhejiang provinces [1]. A recent study reported the presence of *L. aurea* in Shanxi Province [2]. *L. aurea* exhibits significant horticultural and medicinal value. Horticulturally, it serves as an effective groundcover in shaded understory environments and is widely employed in formal flower borders and alpine or rock gardens. It is also cultivated successfully as a container plant and harvested for use as a cut flower. Medicinally, its bulbs are rich in bioactive alkaloids—particularly lycorine and galanthamine—both of which demonstrate well-documented, multifaceted pharmacological activities, including acetylcholinesterase inhibition, antitumor and antiviral effects. Owing to its relatively high galanthamine content and favorable biosynthetic profile, *L. aurea* is recognized as a promising botanical resource for the sustainable production of galanthamine, a compound approved for clinical use in Alzheimer’s disease management [1,3,4].

*L. aurea* is a morphological and cytological species complex, which includes *L. aurea* var. *aurea*, *L. aurea* var. *surgens*, *L. aurea* var. *angustitepala*, *L. traubii* and *L. longifolia* [1,5,6,7,8]. *L. traubii* and *L. aurea* are morphologically similar and closely related, but there is still debate over whether *L. traubii* should be included in the *L. aurea* species complex [1,5,6,7,8,9]. The latest research suggests that *L. longifolia* is a geographical population of *L. aurea* [7]. Among the approximately 20 recognized species of *Lycoris*, *L. aurea* displays the most extensive intraspecific variation in chromosome number, exhibiting dysploid series ranging from 2*n* = 12 to 2*n* = 16 [1,5,6,7,10,11]. Kurita established a foundational chromosomal classification system for the genus *Lycoris*, delineating three basic types based on arm ratio: M-type (metacentric and submetacentric chromosomes, arm ratio = 1.0–3.0), A-type (acrocentric chromosomes, arm ratio = 3.01–19.99), and T-type (telocentric chromosomes characterized by dot-like short arms and arm ratio > 20) [12]. This tripartite system has been widely adopted in cytological and taxonomic studies of *Lycoris*. Prior to this classification system, chromosomes in *Lycoris* were historically categorized into two types: rod (I)-shaped chromosomes (corresponding to the A- and T-type chromosomes) and V-shaped chromosomes (corresponding to the M-type chromosomes) [13]. The reported populations of *L. aurea* include five cytotypes: 2*n* = 12 = 10M + 2T, 2*n* = 13 = 9M + 4T, 2*n* = 14 = 8M + 6T, 2*n* = 15 = 7M + 8T, and 2*n* = 16 = 6M + 10T [5,6,10,11,14,15,16,17,18]. A recent comprehensive cytogeographic survey of the *L. aurea* complex across its entire distribution range in China identified four distinct cytotypes: 2*n* = 12, 2*n* = 14, 2*n* = 15, and 2*n* = 16; no population exhibited 2*n* = 13. Of these, 2*n* = 14 and 2*n* = 16 are the most prevalent cytotypes, whereas 2*n* = 12 is exceedingly rare [6].

Of the approximately 20 taxonomically recognized species in the genus *Lycoris*, more than half are polyploids or sterile hybrids; only nine have been cytologically verified as fertile diploids [1,5]. *Lycoris* displays extensive interspecific and intraspecific variation in both chromosome number (2*n* = 12–44) and karyotype structure, with documented basic chromosome numbers (*x*) of 6, 7, 8, and 11 [7,12,19]. Notably, the total number of long arms (i.e., major arms) per diploid chromosome complement is invariably a multiple of 11 across all currently recognized species—a cytogenetic invariant first documented by Inariyama [20] and subsequently confirmed in numerous studies [5,7,9,10,17,21,22,23,24,25,26,27]. Collectively, these findings indicate that hybridization, polyploidy, and dysploidy are the principal drivers of species diversification and chromosomal evolution in *Lycoris* [5,7,18,21,26,28,29]. Dysploidy refers to evolutionary changes in the basic chromosome number arising from chromosomal structural rearrangements—particularly chromosome fusions or fissions—that alter the number of chromosomal units without significantly changing the overall genomic content [30]. The evolutionary mechanism responsible for dysploidy in the genus *Lycoris*—whether attributable to Robertsonian translocations (centric fusions, associated with descending dysploidy) or centric fissions (associated with ascending dysploidy)—remains unresolved and is the subject of ongoing debate in the cytogenetic literature [5,10,17,20,26,31,32,33,34]. This uncertainty persists primarily due to the absence of cytogenetically conserved chromosomal markers that can unambiguously resolve structural rearrangements.

Karyotype analysis, a foundational cytogenetic method, enables genome characterization at the chromosomal level and facilitates interspecific evolutionary inference through karyotypic comparison, thereby contributing robustly to plant systematics [35,36,37,38]. Conventional karyotype analysis—relying solely on chromosome number and gross morphological features such as length, arm ratio, and satellite presence—is inherently constrained by the paucity of cytologically distinguishable landmarks, resulting in imprecise karyotype analysis and providing limited information about the organization of the genome. Since the 1960s, cytogenetic techniques such as Giemsa banding, fluorochrome banding, and fluorescence in situ hybridization (FISH) have been developed successively. These methods enable high-resolution mapping of heterochromatic regions and specific DNA sequences on chromosomes, thereby furnishing robust, chromosome-specific landmarks essential for accurate karyotyping and genome structural analysis. In plant chromosome characterization, dual-fluorochrome banding techniques, such as CMA (chromomycin A3)/DAPI (4,6-diamidino-2-phenylindole) staining and PI (propidium iodide)/DAPI staining, have been widely adopted. The latter technique—termed CPD staining (combined PI and DAPI staining)—enables simultaneous visualization of AT-rich and GC-rich heterochromatic regions on chromosomes: AT-rich and GC-rich domains appear as blue DAPI-positive bands (hereinafter referred to as DAPI bands) and red CPD bands, respectively [36,39,40]. Critically, its sensitivity for detecting GC-rich 45S rDNA sites is comparable to that of FISH using a 45S rDNA-specific probe [39]. The most commonly used DNA probes in plant molecular cytogenetic analysis are 5S and 45S (18S-5.8S-26S) rDNA sequences. Comparative analysis of fluorochrome banding and rDNA FISH patterns—specifically, the number and chromosomal positions of fluorescent bands and rDNA signals—across taxa within a genus or species enables the detection of genomic differentiation. Such cytogenetic comparisons provide critical insights into evolutionary relationships among the studied taxa and facilitate inference of chromosomal evolutionary trends at the generic or specific level [36,37,38].

Assessment of karyotype asymmetry constitutes a fundamental and essential component of plant karyotypic analysis. Comparative evaluation of karyotype asymmetry across taxa provides valuable insights into karyological affinities and evolutionary trajectories. Over the past several decades, numerous researchers have proposed more than a dozen karyotype asymmetry indices to quantify structural heterogeneity within chromosome complements. Nevertheless, rigorous methodological evaluations—particularly those grounded in statistical robustness and biological interpretability—have identified the Mean Centromeric Asymmetry (M_CA_) index and the Coefficient of Variation of Chromosome Length (CV_CL_) as the most reliable and biologically informative metrics for assessing intrachromosomal asymmetry (i.e., variation in centromere position along individual chromosomes) and interchromosomal asymmetry (i.e., disparity in chromosome size across the complement), respectively [41,42]. The most robust approach for comparing the karyotype asymmetry across taxa involves generating a two-dimensional scatter plot of M_CA_ versus CV_CL_. In contrast, principal coordinates analysis (PCoA) based on six karyotypic parameters—*x*, 2*n*, TCL (total length of haploid complement), CV_CI_ (Coefficient of Variation of Centromeric Index), M_CA_, and CV_CL_—represents the most effective method for inferring karyological relationships among taxa [11,37,38,40,42,43].

Previous cytogenetic studies on *L. aurea* have predominantly relied on chromosome counting and conventional karyotype analysis, with most investigations restricted to a single or only a few geographically limited populations [7,10,15,16,17,19,44,45,46]. The recent comprehensive cytogeographic study by Wang et al. on the *L. aurea* complex—despite encompassing 46 natural populations across China—employed only conventional karyotype analysis [6]. Chang et al. first reported the physical mapping of 5S and 45S rDNA loci in a 2*n* = 14 population of *L. aurea* native to Taiwan Island [33]. To date, only four additional molecular cytogenetic studies have been published on this species: Quan et al. performed CPD staining and FISH mapping of 5S and 45S rDNA sites in a 2*n* = 14 population from Yuanling County, Hunan Province [18]; Zhang et al. established 5S/45S rDNA FISH-based karyotypes for two distinct populations, one 2*n* = 14 and another 2*n* = 16 [26,27]; and Jiang et al. conducted CPD staining and dual-color 5S/45S rDNA FISH across eight natural populations—comprising four 2*n* = 14, two 2*n* = 15, and two 2*n* = 16 [11]. In the third article, our group reported inter-population variation in fluorochrome banding and rDNA FISH patterns in *L. aurea*—providing the first cytological evidence of divergence associated with cytotype differentiation [11]. However, as the study prioritized methodological development and validation, and its sampling encompassed only a limited number of populations, it did not permit a comprehensive assessment of heterochromatin differentiation and the evolutionary dynamics of rDNA sites across distinct cytotypes as well as across populations sharing the same cytotype.

In this study, we performed cytogenetic characterization of 20 natural populations of the *L. aurea* complex—collected across diverse geographic regions of China—using CPD staining and FISH with 5S and 45S rDNA probes. By integrating quantitative data on chromosome morphology, fluorescent bands and rDNA FISH signals, we established standardized quantitative molecular cytogenetic karyotypes for each population. Karyotype asymmetry was assessed using the widely recognized asymmetry indices and inter-population karyological relationships were inferred using six karyotypic parameters. We also conducted a geographical differentiation analysis for the three prevalent cytotypes identified in natural populations of the *L. aurea* complex by integrating collection site data from the present study with those reported in the literature. Our analyses comprehensively delineate chromosomal-level genomic differentiation and karyotypic diversity both among cytotypes and within cytotype-specific population lineages. Furthermore, we propose a working hypothesis regarding the mechanisms underlying dysploidy in the *L. aurea* complex through comparative karyotypic analysis across cytotypes, with particular attention to the patterns of AT-rich heterochromatic regions and rDNA sites. Furthermore, integrating cytogenetic evidence, cytotype geographical distribution patterns, and recently published paleoclimatic ecological niche modeling (ENM) results, we propose a hypothesis-generating, integrative evolutionary biogeographic framework for this species complex.

## 2. Results

### 2.1. General Karyotypic Characteristics

**Figure 1:**
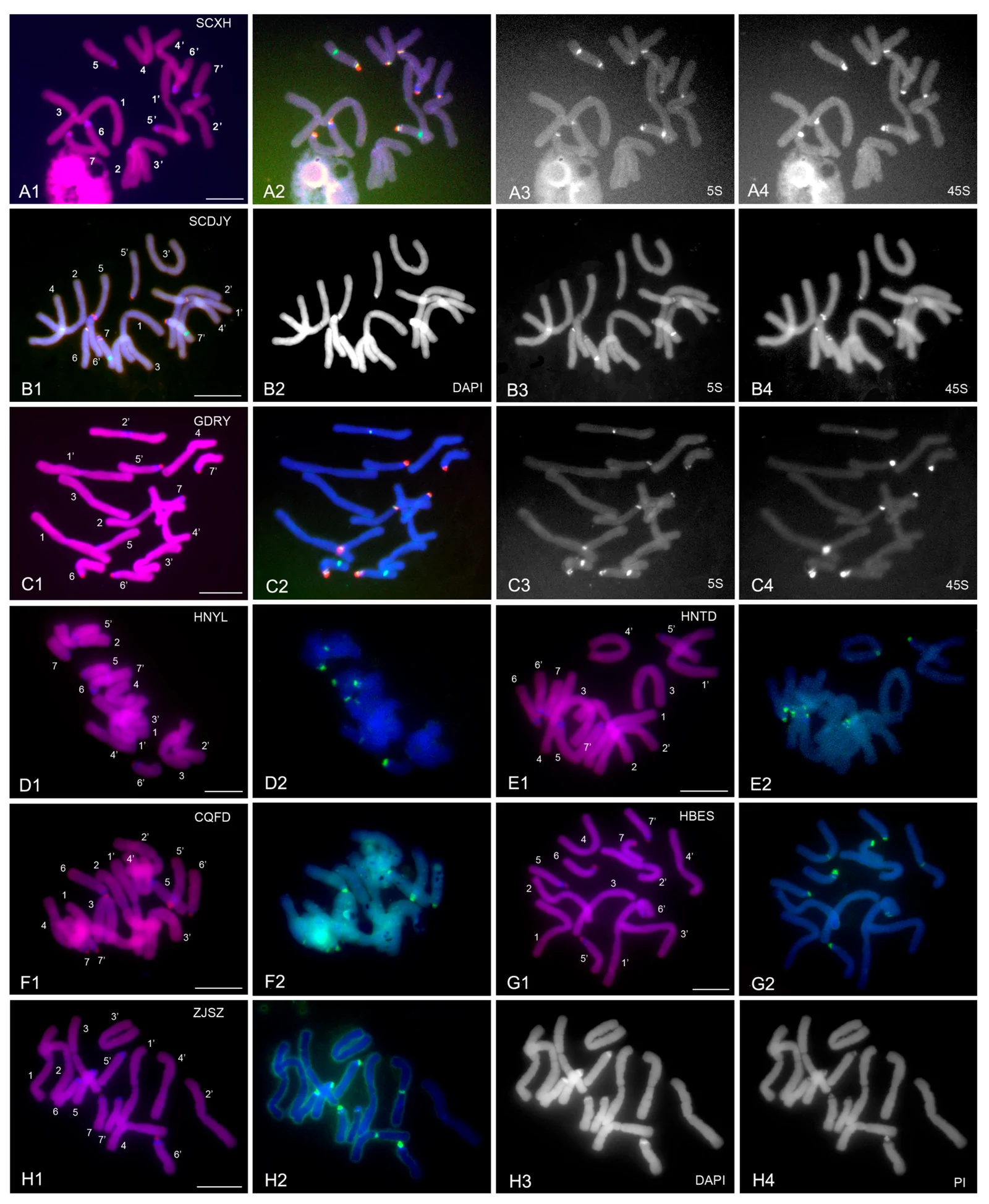
Mitotic metaphase chromosomes of 2*n* = 14 (cytotype A) populations within the *Lycoris aurea* complex, visualized by fluorochrome banding and FISH with rDNA probes. (**A**–**H**) denote the SCXH, SCDJY, GDRY, HNYL, HNTD, CQFD, HBES, and ZJSZ populations, respectively. (**A1**,**C1**,**D1**,**E1**,**F1**,**G1**,**H1**) illustrate chromosomes subjected to CPD staining, revealing red CPD bands and blue DAPI bands. (**A2**,**B1**,**C2**) depict chromosomes hybridized with dual-color FISH using 5S rDNA (green) and 45S rDNA (red) oligonucleotide probes; total DNA was counterstained with DAPI. (**A3**,**B3**,**C3**) are grayscale micrographs displaying 5S rDNA hybridization signals; (**A4**,**B4**,**C4**) are grayscale micrographs displaying 45S rDNA hybridization signals; and (**B2**) shows the DAPI-stained chromosomes as a grayscale micrograph. (**D2**,**E2**,**F2**,**G2**,**H2**) represent chromosomes hybridized with mono-color FISH using 45S rDNA probe (green); total DNA was counterstained with DAPI. (**H3**,**H4**) are grayscale micrographs showing DAPI- and PI-stained chromosomes, respectively, following CPD staining. Chromosome numbering follows karyotype analysis. The population name is indicated in the upper-right corner of the first image of each population. Bar = 10 µm.

**Figure 2:**
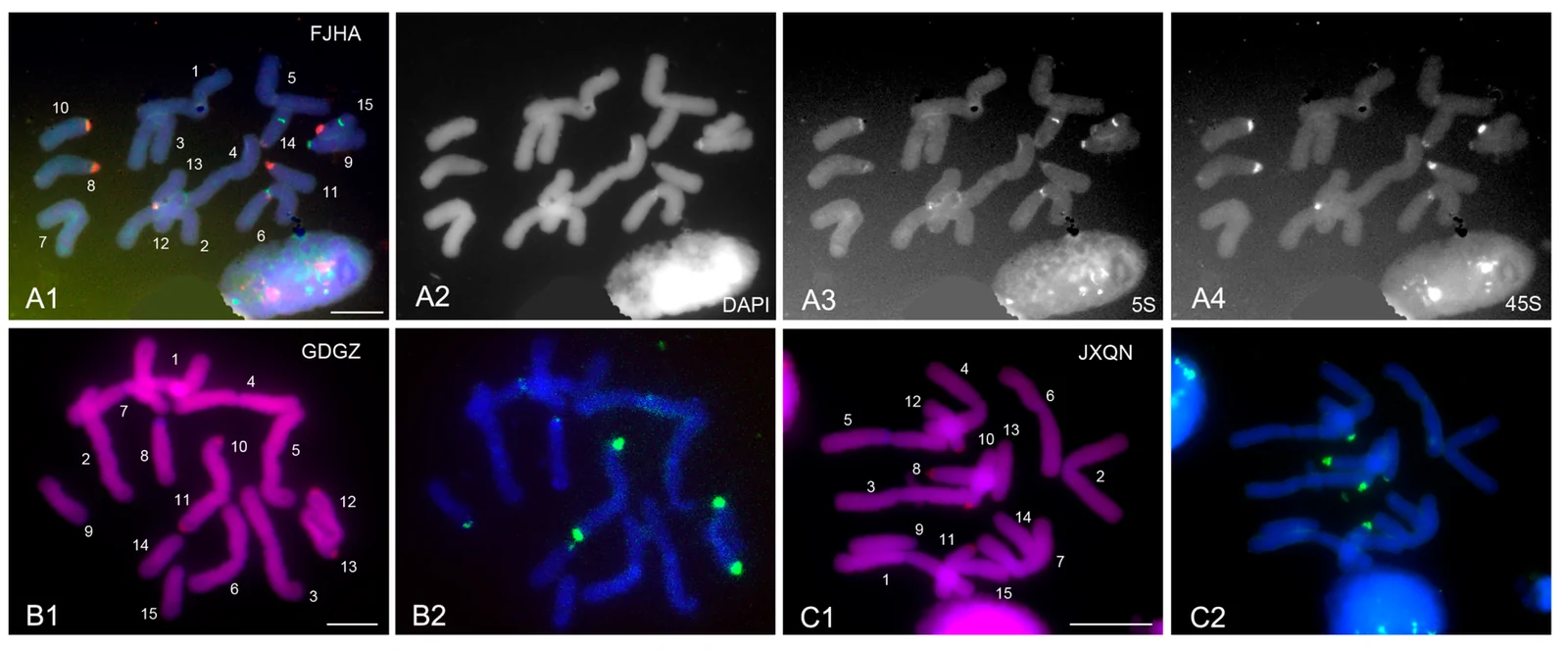
Mitotic metaphase chromosomes of 2*n* = 15 (cytotype B) populations within the *Lycoris aurea* complex, visualized by fluorochrome banding and FISH with rDNA probes. (**A**–**C**) denote the FJHA, GDGZ, and JXQN populations, respectively. (**A1**) shows chromosomes hybridized with dual-color FISH using 5S rDNA (green) and 45S rDNA (red) oligonucleotide probes; total DNA was counterstained with DAPI. (**A2**–**A4**) are grayscale micrographs displaying DAPI-stained chromosomes, 5S rDNA hybridization signals, and 45S rDNA hybridization signals, respectively. (**B1**,**C1**) illustrate chromosomes subjected to CPD staining, revealing red CPD bands and blue DAPI bands. (**B2**,**C2**) depict chromosomes hybridized with mono-color FISH using 45S rDNA probe (green); total DNA was counterstained with DAPI. Chromosome numbering follows karyotypic analysis. The population name is indicated in the upper-right corner of the first image of each population. Bar = 10 µm.

**Figure 3:**
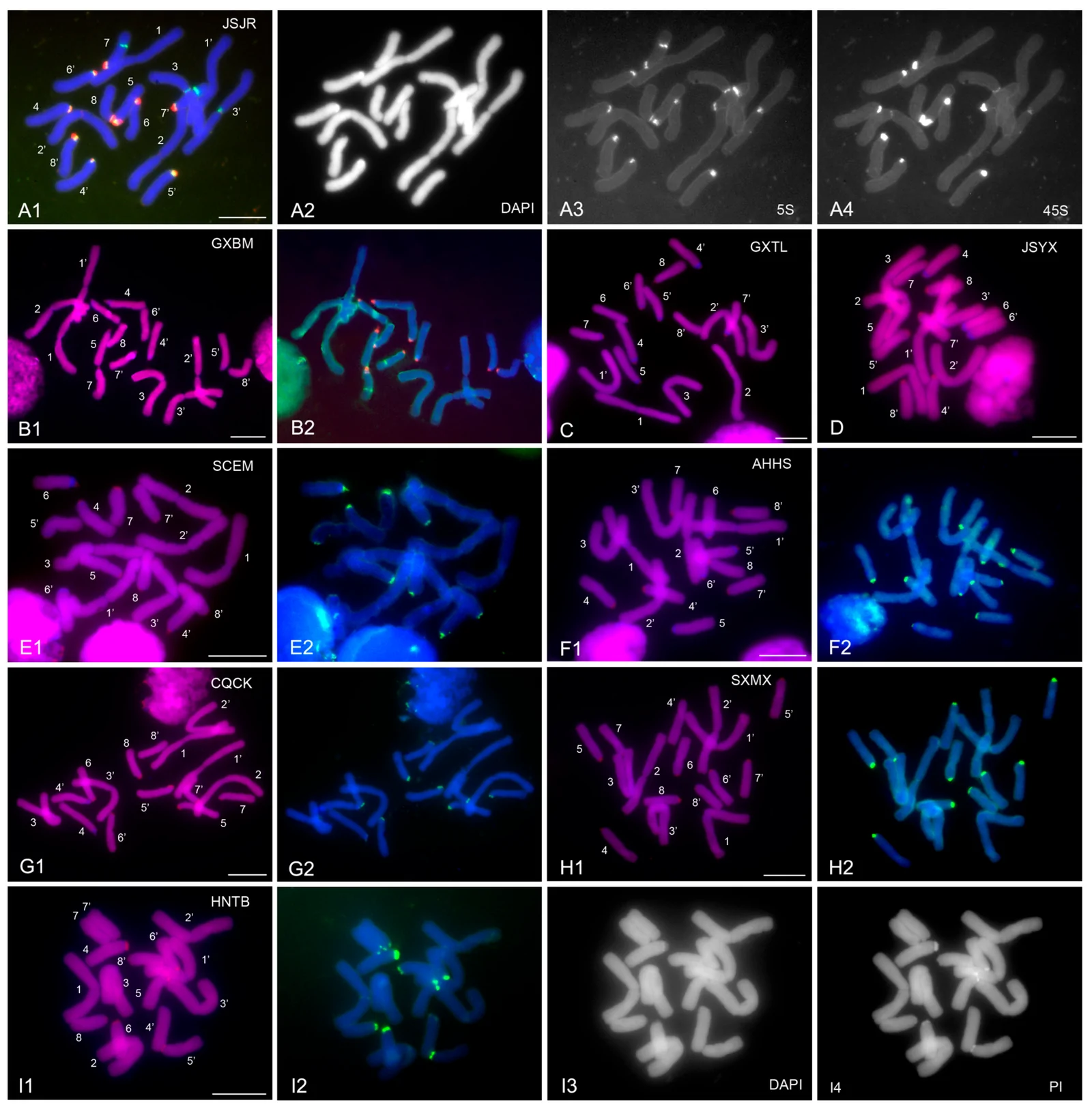
Mitotic metaphase chromosomes of 2*n* = 16 (cytotype C) populations within the *Lycoris aurea* complex, visualized by fluorochrome banding and FISH with rDNA probes. (**A**–**I**) denote the JSJR, GXBM, GXTL, JSYX, SCEMS, AHHS, CQCK, SXMX, and HNTB populations, respectively. (**B1**,**C**,**D**,**E1**,**F1**,**G1**,**H1**,**I1**) illustrate chromosomes subjected to CPD staining, revealing red CPD bands and blue DAPI bands. (**A1**,**B2**) depict chromosomes hybridized with dual-color FISH using 5S rDNA (green) and 45S rDNA (red) oligonucleotide probes; total DNA was counterstained with DAPI. (**A2**–**A4**) are grayscale micrographs displaying DAPI-stained chromosomes, 5S rDNA hybridization signals, and 45S rDNA hybridization signals, respectively. (**E2**,**F2**,**G2**,**H2**,**I2**) represent chromosomes hybridized with mono-color FISH using 45S rDNA probe (green); total DNA was counterstained with DAPI. (**I3**,**I4**) are grayscale micrographs showing DAPI- and PI-stained chromosomes, respectively, following CPD staining. Chromosome numbering follows karyotype analysis. The population name is indicated in the upper-right corner of the first image of each population. Bar = 10 µm.

**Figure 4:**
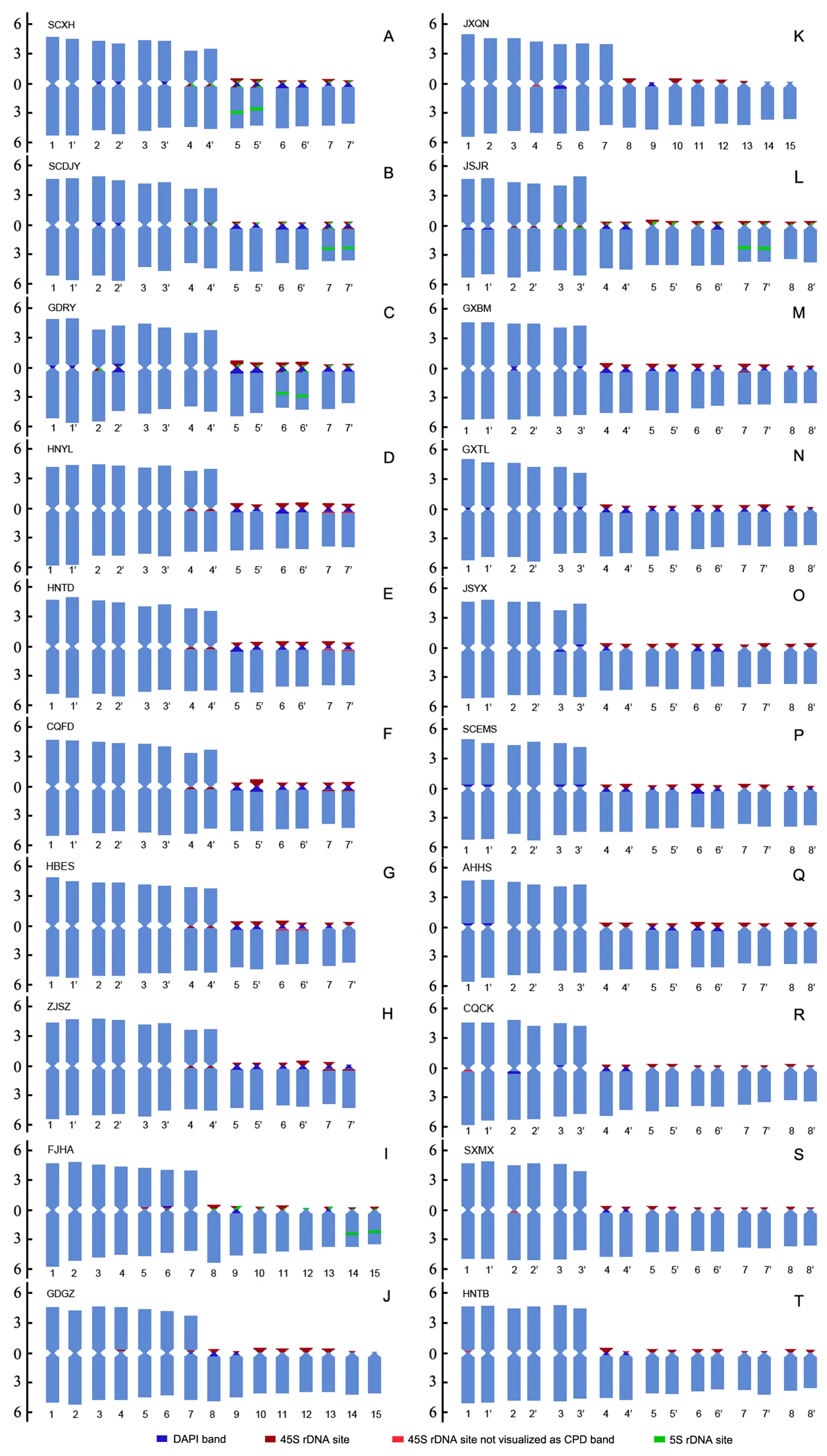
Idiograms of twenty populations of the *Lycoris aurea* complex, illustrating the chromosome measurements, and the position and size of fluorescent bands and rDNA FISH signals. (**A**) SCXH; (**B**) SCDJY; (**C**) GDRY; (**D**) HNYL; (**E**) HNTD; (**F**) CQFD; (**G**) HBES; (**H**) ZJSZ; (**I**) FJHA; (**J**) GDGZ; (**K**) JXQN; (**L**) JSJR; (**M**) GXBM; (**N**) GXTL; (**O**) JSYX; (**P**) SCEMS; (**Q**) AHHS; (**R**) CQCK; (**S**) SXMX; (**T**) HNTB. The left ordinate indicates relative chromosome length as a percentage of the total diploid chromosome length (TDL). Chromosome serial numbers are annotated at the base of each idiogram. Chromosomes are arranged in descending order of length: for 2*n* = 14 and 2*n* = 16 populations, homologous pairs are ordered by decreasing pair length; for 2*n* = 15 populations, chromosomes are ordered individually by decreasing length. The population name is indicated in the upper-left corner of each corresponding ideogram.

Eight karyotypic characteristics—including karyotype formula, the total diploid chromosome length (TDL), range of chromosome relative length (RRL), mean centromeric index (CI), CV_CI_, M_CA_, CV_CL_ and Stebbins’ type—were determined for all 20 populations studied and are summarized in Table 1. Representative metaphase chromosomes for these populations are presented in Figure 1, Figure 2 and Figure 3, and corresponding standardized karyotype ideograms are provided in Figure 4.

Karyotype analysis of somatic metaphase cells across the 20 *L. aurea* populations revealed three distinct chromosome numbers (Table 1; Figure 1, Figure 2 and Figure 3): 2*n* = 14 in eight populations, 2*n* = 15 in three populations, and 2*n* = 16 in nine populations. This intraspecific variation in somatic chromosome number constitutes dysploidy. No B chromosomes were observed in any of the 20 populations examined. For clarity and consistency in subsequent analyses and discussion, the *L. aurea* populations characterized by somatic chromosome numbers of 2*n* = 14, 2*n* = 15, and 2*n* = 16 are designated cytotype A, cytotype B, and cytotype C, respectively. The TDLs varied across populations of cytotypes A, B, and C, with respective ranges of 184.74–236.23 µm, 171.96–259.78 µm, and 164.64–273.09 µm. To assess whether TDL varied significantly across the three cytotypes, we conducted a one-way analysis of variance (ANOVA) on all 20 population-level TDL measurements (cytotype A: *n* = 8; cytotype B: *n* = 3; cytotype C: *n* = 9). Prior to ANOVA, normality and homoscedasticity were rigorously evaluated using the Shapiro–Wilk test and Levene’s test, respectively; both assumptions were satisfied (all *p* > 0.05). The ANOVA revealed no statistically significant difference in TDL among the three cytotypes (F(2, 17) = 0.481, *p* = 0.626, η^2^ = 0.054, 95%, CI = 0.000–0.289).

The karyotypes of the *L. aurea* populations exhibit pronounced bimodality, characterized by a distinct size dichotomy between large metacentric chromosomes (m) and relatively smaller telocentric (t) and subtelocentric (st) chromosomes (Table 1 and Table S2; Figure 4). The centromere positions of t- and st-chromosomes in *L. aurea* are often ambiguous due to the absence of a clearly discernible primary constriction. Conventional karyotyping methods—based on staining with conventional dyes or DAPI—have been extensively employed in previous studies. However, the 45S rDNA sites located on the short arms of the t- and st-chromosomes [33] are GC-rich and consequently exhibit weak or absent staining under standard cytological protocols (Figure 1(H3,H4) and Figure 3(I3,I4)). This staining deficiency impedes reliable centromere identification and may result in systematic underestimation of short-arm length. In this study, the terminal regions of t- and st-chromosomes exhibiting red bands after CPD staining or 45S rDNA hybridization signals after FISH were incorporated into short-arm length measurements—these regions account for the predominant portion of the respective short arm. We therefore inferred that the centromere resides at the interface between the rDNA chromatin domain and the adjacent chromosome segment. This inference is supported by the clear visualization of a primary constriction at this interface in several metaphase chromosomes (e.g., Figure 1(G1) and Figure 3(G1,H1,I1)). For t- or st-chromosomes displaying DAPI bands, the centromere is positioned at the DAPI-band side of the DAPI–CPD band junction: specifically, a minor fraction of the DAPI signal overlaps the centromere, whereas the majority corresponds to the pericentromeric region of the long arm. In the rare cases where the short arm lacks detectable 45S rDNA, the centromere was operationally defined as the subtly constricted zone near the chromosome terminus (e.g., chromosome 7′ in Figure 1(H1); chromosome 15 in Figure 2(B1); chromosomes 14 and 15 in Figure 2(C1)). Following centromere localization and precise measurement of short- and long-arm lengths according to the criteria outlined above, we found that chromosomes with an arm ratio exceeding 20 are absent or rare across all examined populations (Table S2). Consequently, Kurita’s chromosomal classification scheme for the genus *Lycoris* is no longer applicable to *L. aurea* [12]. Accordingly, this study adopts the classical chromosome nomenclature system of Levan et al. [47] for karyotypic characterization of the *L. aurea* complex (Table 1 and Table S2). In cytotype A, four populations (SCXH, SCDJY, HNTD, and ZJSZ) exhibit a karyotype formula of 2*n* = 14 = 8m + 6t; the remaining four populations (GDRY, HNYL, CQFD, and HBES) display 2*n* = 14 = 8m + 1st + 5t. In cytotype B, FJHA and JXQN share the formula 2*n* = 15 = 7m + 8t, whereas GDGZ exhibits 2*n* = 15 = 7m + 2st + 6t. In cytotype C, JSJR is the sole exception, with a karyotype formula of 2*n* = 16 = 6m + 1st + 9t; all other eight populations (GXBM, GXTL, JSYX, SCEMS, AHHS, CQCK, SXMX, and HNTB) conform to 2*n* = 16 = 6m + 10t (Table 1 and Table S2).

In most populations of cytotypes A and C, the two members of certain chromosome pairs exhibit pronounced heteromorphism with respect to length and/or arm ratio: chromosome pairs 2–5 in SCXH (Figure 1(A1–A4) and Figure 4A); chromosome pairs 1–4 and 6 in SCDJY (Figure 1(B1–B4) and Figure 4B); chromosome pairs 1–7 in GDRY (Figure 1(C1–C4) and Figure 4C); chromosome pairs 4 and 7 in CQFD (Figure 1(F1,F2) and Figure 4F); chromosome pairs 5 and 7 in HBES (Figure 1(G1,G2) and Figure 4G); chromosome pairs 1, 3, 6, and 7 in ZJSZ (Figure 1(H1–H4) and Figure 4H); chromosome pairs 2, 3, and 8 in JSJR (Figure 3(A1–A4) and Figure 4L); chromosome pair 5 in GXBM (Figure 3(B1,B2) and Figure 4M); chromosome pairs 1–5 and 8 in GXTL (Figure 3C and Figure 4N); chromosome pairs 3 and 5–7 in JSYX (Figure 3D and Figure 4O); chromosome pairs 2, 3, and 7 in SCEMS (Figure 3(E1,E2) and Figure 4P); chromosome pair 7 in AHHS (Figure 3(F1,F2) and Figure 4Q); chromosome pairs 2, 4, 5, and 8 in CQCK (Figure 3(G1,G2) and Figure 4R); chromosome pair 3 in SXMX (Figure 3(H1,H2) and Figure 4S); and chromosome pairs 4, 7, and 8 in HNTB (Figure 3(I1–I4) and Figure 4T). Chromosomal morphological differentiation in cytotype B populations is more pronounced (Figure 2(A1–C2) and Figure 4I–K), precluding reliable chromosome pairing based on morphological similarity only.

The mean centromere index (CI)—a quantitative metric that reflects interchromosomal variation in centromere position across a karyotype—shows only slight variation among populations sharing the same cytotype. In contrast, significant inter-cytotypic divergence is observed: CI values decline progressively from cytotype A (30.20 ± 19.86~31.67 ± 19.65), through cytotype B (25.84 ± 20.98~26.89 ± 20.69), to cytotype C (22.26 ± 19.64~24.66 ± 18.81) (Table 1). This consistent gradient implies that centromere index variability is highest in cytotype C, intermediate in cytotype B, and lowest in cytotype A. The M_CA_ value—which quantifies intrachromosomal asymmetry within a chromosome complement—the CV_CL_ value—which reflects interchromosomal asymmetry—and the CV_CI_ value—which measures centromere position heterogeneity—all vary among populations of the same cytotype and displayed a consistent stepwise increase from cytotype A (M_CA_: 36.67–39.60; CV_CL_: 31.23–35.78; CV_CI_: 57.88–69.25) to cytotype B (M_CA_: 46.21–48.33; CV_CL_: 35.78–38.32; CV_CI_: 76.95–81.21), and further to cytotype C (M_CA_: 50.67–55.48; CV_CL_: 39.81–44.84; CV_CI_: 76.25–94.09) (Table 1). According to Stebbins’ karyotype asymmetry category, the analyzed populations exhibited graded levels of karyotypic asymmetry: cytotype A was assigned to type 2B, while cytotypes B and C were both classified as type 3B —indicating a moderate to high degree of karyotypic asymmetry in the *L. aurea* complex (Table 1).

**Figure 5:**
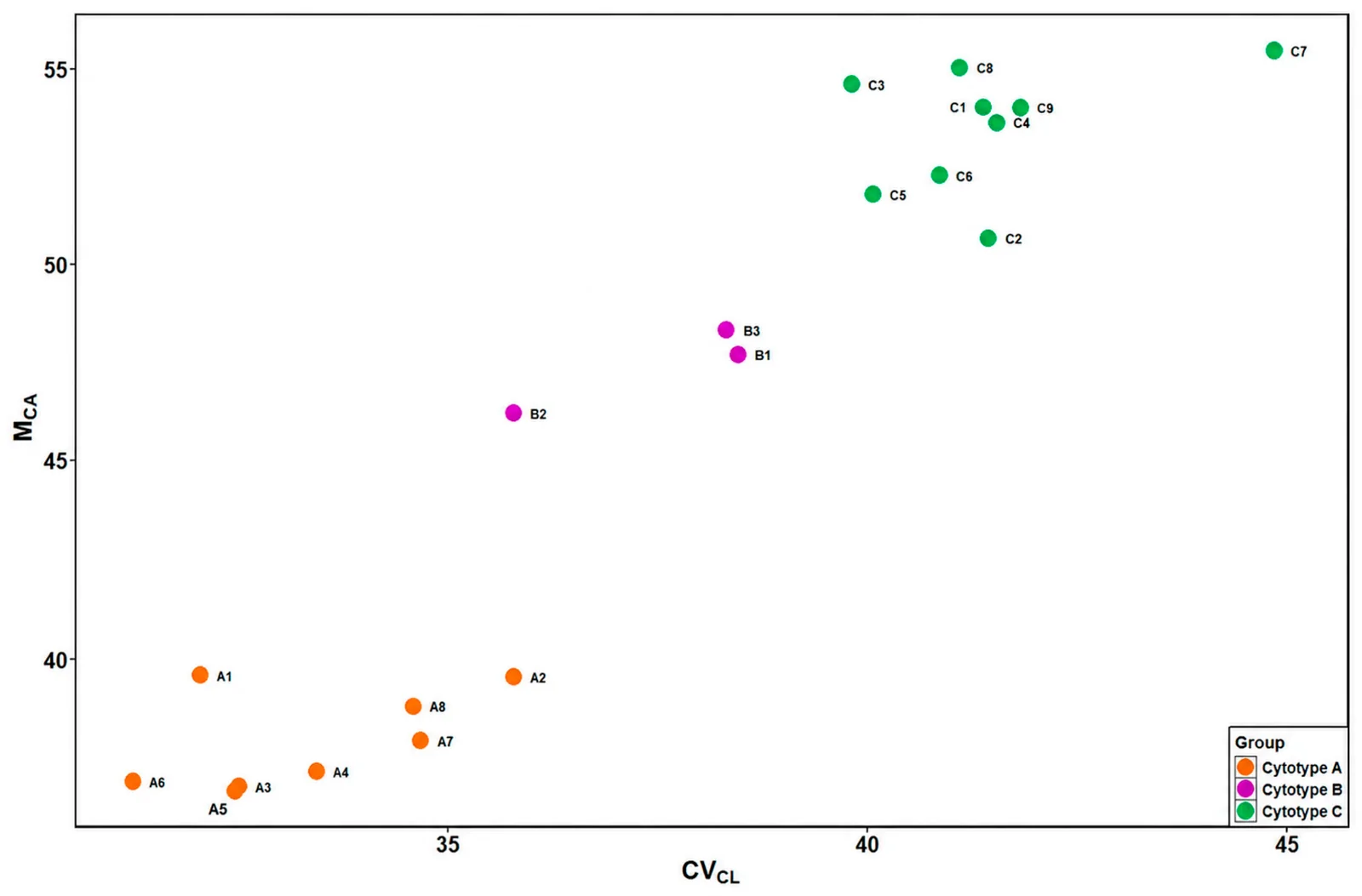
Bidimensional scatter plot of M_CA_ versus CV_CL_ for 20 populations of the *Lycoris aurea* complex. A1–A8 denote the SCXH, SCDJY, GDRY, HNYL, HNTD, CQFD, HBES, and ZJSZ populations, respectively, all belonging to cytotype A (2*n* = 14). B1–B3 denote the FJHA, GDGZ, and JXQN populations, respectively, all belonging to cytotype B (2*n* = 15). C1–C9 denote the GXBM, JSJR, GXTL, SCEM, AHHS, JSYX, CQCK, SXMX, and HNTB populations, respectively, all belonging to cytotype C (2*n* = 16).

The two-dimensional scatter plot of M_CA_ versus CV_CL_ fully discriminates the karyotypic structures of all 20 populations, with no inter-population overlap (Figure 5). Furthermore, the plot partitions these populations into three well-defined, non-overlapping clusters that correspond precisely to cytotypes A, B, and C, visually reflecting a progressive increase in both intrachromosomal and interchromosomal asymmetry across the cytotype series from A to B to C.

**Figure 6:**
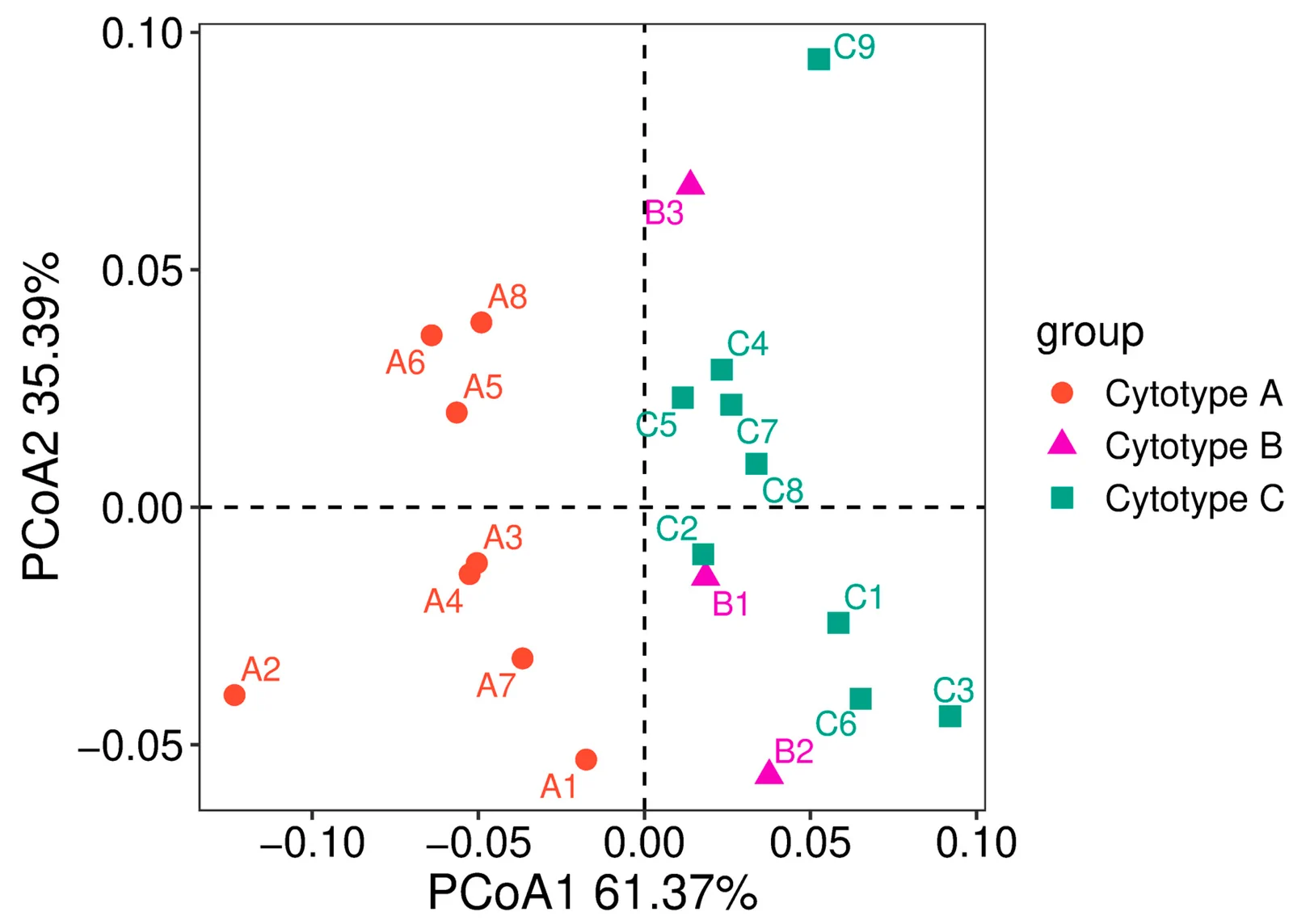
Principal coordinate analysis (PCoA) of 20 populations of the *Lycoris aurea* complex based on six karyotypic parameters: *x*, 2*n*, TDL, M_CA_, CV_CL_ and CV_CI_. Population labels correspond to those in Figure 5. The vertical and horizontal dotted lines indicate the PCoA1 (x-axis) and PCoA2 (y-axis) coordinate axes, respectively. The first principal coordinate (PCoA1) explains 61.37% of the total variance and captures the dominant pattern of karyotypic differentiation; the second principal coordinate (PCoA2) accounts for an additional 35.39%, reflecting secondary axes of divergence. Together, PCoA1 and PCoA2 cumulatively explain 96.76% of the total variance, indicating that the two-dimensional ordination effectively retains the major structuring signals of the original multivariate karyotypic dataset.

Principal coordinate analysis (PCoA) based on six karyotypic parameters—*x*, 2*n*, TDL, CV_CI_, M_CA_, and CV_CL_—for 20 populations accounted for 96.76% of the total variation, with PCoA1 and PCoA2 explaining 61.37% and 35.39%, respectively. The resulting two-dimensional ordination plot reveals clear separation among the three cytotypes (Figure 6). PCoA ordination separated the 20 populations into two well-delineated clusters along the PCoA1 axis (X-axis): the eight populations of cytotype A formed a distinct leftward cluster, whereas the three populations of cytotype B and nine of cytotype C jointly constituted a rightward cluster. All cytotype A samples were tightly aggregated along the negative PCoA1 axis, exhibiting complete spatial separation from cytotypes B and C; this pattern reflects a compact and karyotypically distinct cluster. Furthermore, within cytotype A, samples exhibited consistent bimodal partitioning along the PCoA2 axis (Y-axis), resolving into two spatially discrete subclusters. The three cytotype B samples are positioned in the vicinity of the ordination origin, occupying an intermediate position between the two dominant clusters—cytotype A (left) and cytotype C (right). All cytotype C samples are aligned along the positive PCoA1 axis, collectively occupying the overwhelming majority of the rightward ordination space. They display broad dispersion and a pronounced intra-cytotypic differentiation gradient along the PCoA2 axis (Y-axis), resolving into two spatially distinct subclusters. To assess whether this pattern was influenced by variables intrinsically linked to cytotype designation or exhibiting high redundancy, we evaluated the sensitivity of the PCoA ordination to variable selection. Given that *x* and 2*n* are intrinsically linked to cytotype designation and that CV_CI_ exhibited strong correlation with both M_CA_ and CV_CL_, a sensitivity PCoA ordination was conducted excluding these three variables (*x*, 2*n*, and CV_CI_). The PCoA ordination based on TDL, M_CA_, and CV_CL_ accounted for 70.64% and 18.71% of the total variation along PCoA1 and PCoA2, respectively (cumulatively 89.35%), and preserved the overall ordination pattern: cytotype A remained distinct from cytotypes B and C, whereas cytotype B occupied an intermediate position between them (Figure S1). Similar ordination patterns were observed in additional sensitivity analyses conducted with the variable combinations TDL + MCA and TDL + CVCL. Thus, the primary pattern of karyotypic differentiation identified in the original six-variable PCoA ordination was preserved following exclusion of the chromosome-number variables (*x* and 2*n*) and CV_CI_, indicating that the ordination structure was not attributable solely to cytotype-defining variables or to statistically redundant measures of karyotypic asymmetry.

**Figure 7:**
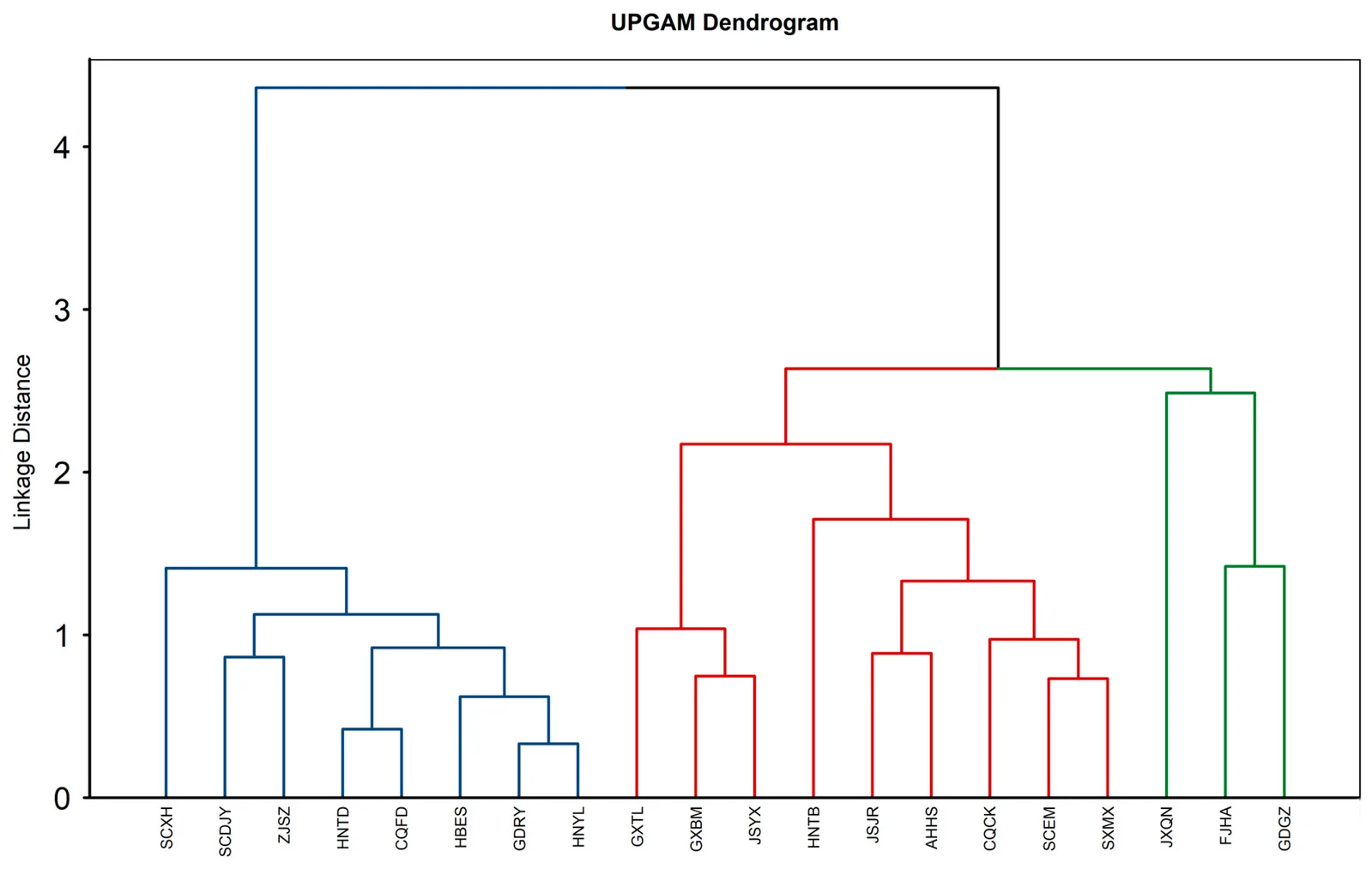
UPGMA dendrogram depicting hierarchical clustering and karyotypic similarity among 20 populations of the *Lycoris aurea* complex, constructed using six karyotypic parameters: *x*, 2*n*, TDL, M_CA_, CV_CL_, and CV_CI_. Blue, green, and red lines depict the hierarchical clustering topologies of the cytotype A, cytotype B, and cytotype C populations, respectively. The linkage karyotypic distances represent differences in the multivariate karyotypic features and should not be interpreted as phylogenetic distances.

The UPGMA dendrogram, generated based on six karyotypic parameters—*x*, 2*n*, TDL, CV_CI_, M_CA_, and CV_CL_—partitioned the 20 populations into two major clusters (Figure 7). The first cluster encompassed all eight populations assigned to cytotype A, while the second cluster included all populations of cytotypes B and C. These two major clusters converged at a relatively high karyotypic linkage distance of approximately 4.3, reflecting substantial differentiation in overall karyotypic architecture between cytotype A and the combined cytotypes B and C. Within cytotype A, all eight populations clustered within a karyotypic linkage distance of approximately 1.5, indicating high intra-cytotypic karyotypic uniformity and low inter-population differentiation. Within the second major cluster, cytotypes B and C formed two well-defined subclusters that coalesced at a linkage distance of approximately 2.6–2.7, suggesting greater karyotypic similarity between these two cytotypes than either share with cytotype A. Notably, cytotype C exhibited a more branched and spatially dispersed clustering topology compared to cytotype A—consistent with elevated intra-cytotypic karyotypic polymorphism. Within cytotype B, populations FJHA and GDGZ formed a tightly resolved subcluster at a karyotypic linkage distance of approximately 1.4, while population JXQN joined this subcluster at a higher linkage distance of approximately 2.5—indicating stronger karyotypic similarity between FJHA and GDGZ and relatively stronger karyotypic differentiation in JXQN.

### 2.2. Fluorochrome Banding and rDNA FISH Patterns

Mitotic metaphase chromosomes of the investigated *L. aurea* populations—visualized by fluorochrome banding and rDNA mapping via FISH—are presented in Figure 1, Figure 2 and Figure 3. The number and position of fluorescent bands and rDNA sites are summarized in Table 2 and illustrated in Figure 4; notably, the number and position of 5S rDNA signal are omitted from Table 2 but are documented in Figure 4. A total of 17 populations were subjected to CPD staining, and all exhibited distinct red CPD bands on somatic chromosomes. Among these, fourteen populations (HNYL, HNTD, CQFD, HBES, ZJSZ, GDGZ, JXQN, GXTL, JSYX, SCEMS, AHHS, CQCK, SXMX, and HNTB) were underwent CPD staining prior to 45S rDNA FISH; the remaining three (SCXH, GDRY, and GXBM) were processed with CPD staining following dual-color FISH with both 5S and 45S rDNA probes. DAPI banding was performed in all 20 populations. Of these, 14 populations exhibited DAPI bands following CPD staining carried out prior to 45S rDNA FISH; the remaining six displayed DAPI bands under alternative experimental protocols: three (SCXH, GDRY, and GXBM) after CPD staining applied post-dual-color FISH, and three (SCDJY, FJHA, and JSJR) after DAPI counterstaining performed subsequent to dual-color FISH. Among the 15 populations for which both CPD bands and 45S rDNA FISH signals were available, all observed CPD banded regions colocalized with 45S rDNA hybridization signals, indicating a strong spatial correspondence between CPD-banded regions and 45S rDNA sites in these materials. Consequently, in the GXTL and JSYX populations—where 45S rDNA FISH signals were unavailable—the observed CPD bands were interpreted as their putative 45S rDNA sites and included in subsequent 45S rDNA site-based analyses. A small subset of 45S rDNA sites—owing to their diminutive size—failed to yield discernible CPD bands after CPD staining (Figure 4; Table 2). For the three populations—SCDJY, FJHA, and JSJR—for which CPD band data were unavailable, the proportion of 45S rDNA chromatin in the TDL was estimated by quantifying the size of 45S rDNA FISH signals. In the dual-color FISH analysis of GXBM, hybridization of the 5S rDNA probe was suboptimal, yielding only faint and sporadic signals—including a single pair of discernible signals in the interstitial regions of the long arms of chromosome pair 7—insufficient for unambiguous locus assignment; thus, 5S rDNA sites were omitted from the karyotype ideogram.

In the eight cytotype A populations, the diploid chromosome complements exhibited 5–9 DAPI bands, 8–10 CPD bands, and 9–10 45S rDNA sites (Figure 1 and Figure 4; Table 2). The cytotype A populations exhibit four consistent cytogenetic features in their fluorochrome banding and rDNA FISH patterns: (1) All three t(st)-chromosome pairs display (CEN + L-PCEN)-DAPI bands (located in both the centromere and the pericentromeric regions of the long arms) with the exception of HBES, which lacks a DAPI band on one member of chromosome pair 7. (2) One pair of m-chromosomes bears CEN- or S/L-PCEN-45S rDNA sites (located in centromere or pericentromeric regions of the short or long arms). (3) 45S rDNA sites are present on the short arms of all three t(st)-chromosome pairs, except in ZJSZ, where the short arm of one member of pair 7 is devoid of such site. (4) One t(st)-chromosome pair carries L-PROX-45S rDNA sites (located in the proximal regions of the long arms). Moreover, dual-color FISH analysis of SCXH, SCDJY, and GDRY reveals a conserved pattern of twelve 5S rDNA sites: ten of these co-localize with 45S rDNA sites (though 5S and 45S signals show partial, not complete, overlap; see Figure 1(A2–A4,B1,B3–B4,C2–C4)), while the remaining two reside in the interstitial regions of the long arms (L-INT) of the t(st)-chromosome pair bearing the L-PROX-45S rDNA sites—serving as a diagnostic cytogenetic marker for this specific chromosome pair. Nonetheless, inter-populational polymorphism is evident in fluorochrome banding and 45S rDNA FISH patterns. Specifically, such variation encompasses differences in the total amounts of DAPI bands and 45S rDNA chromatin, the presence or absence, and number of CEN-DAPI bands on m-chromosomes, and the complete absence of DAPI bands or 45S rDNA sites on certain t-chromosomes in specific populations (Table 2 and Table S2; Figure 4). Moreover, heterozygosity—manifested as inter-homolog differences in 45S rDNA signal intensity and locus size—is consistently observed in certain t-chromosome pairs across all eight populations. In addition, geographically proximate populations display greater congruence in DAPI banding and rDNA FISH patterns—exemplified by the SCXH and SCDJY populations (Figure 1(A1–B4) and Figure 4A,B) and the HNYL and HNTD populations (Figure 1(D1–E2) and Figure 4D,E).

The diploid complements of the three populations assigned to cytotype B exhibit 2–4 DAPI bands, 5–8 CPD bands, and 6–9 45S rDNA sites (Figure 2 and Figure 4; Table 2). FJHA and GDGZ display highly congruent DAPI band and 45S rDNA FISH patterns: (i) both possess a single m-chromosome bearing a CEN-DAPI band, and two t-chromosomes each carrying a (CEN + L-PCEN)-DAPI band; (ii) both harbor two S-PCEN-45S rDNA sites on two m-chromosomes—one colocalized with the CEN-DAPI band—and seven 45S rDNA sites on the short arms of seven t (st)-chromosomes (Figure 2(A1–B2) and Figure 4I,J; Table 2). JXQN exhibits marked cytogenetic divergence from both FJHA and GDGZ: (i) only a single m-chromosome and a single t-chromosome bear L-PROX- and (CEN + L-PCEN)-DAPI bands, respectively; (ii) Only a single m-chromosome—lacking any DAPI band—harbors a L-PCEN-45S rDNA site, and five t-chromosomes bear 45S rDNA sites on their short arms (Figure 2(C1,C2) and Figure 4K; Table 2). Dual-color FISH analysis of FJHA revealed a total of 11 5S rDNA sites (Figure 2(A1,A3,A4) and Figure 4I), distributed as follows: eight sites colocalize with 45S rDNA sites—specifically in the centromeric region of one m-chromosome and on the short arms of seven t-chromosomes—though 5S and 45S signals typically exhibit only partial overlap; one site resides on the short arm of a t-chromosome lacking any 45S rDNA site; and two sites are positioned in the interstitial regions of the long arms of two t-chromosomes. These two t-chromosomes bearing L-INT-5S rDNA sites correspond to the L-INT-5S rDNA-bearing chromosome pair observed in cytotype A populations (Figure 4A–C). Notably, however, unlike in cytotype A, these two t-chromosomes in FJHA lacks the L-PROX-(45S + 5S) rDNA sites.

The diploid chromosome complements of the nine populations of cytotype C exhibit 2–14 DAPI bands, 9–10 CPD bands, and 10–14 45S rDNA sites, showing greater inter-population variability in fluorochrome banding and rDNA FISH patterns compared to cytotype A (Figure 3 and Figure 4L–T; Table 2). The sole consistent cytogenetic feature shared across all nine populations is the presence of a 45S rDNA site on the short arm of each of the ten t(st)-chromosomes. Inter-populational variability primarily reflected in the following cytogenetic features: (i) the total amount of DAPI bands and 45S rDNA chromatin; (ii) the presence/absence, and number of CEN- or PCEN-DAPI bands on m-chromosomes; (iii) the number of (CEN + L-PCEN)-DAPI bands on t-chromosomes; and (iv) the presence/absence, and number of CEN- or S/L-PCEN-45S rDNA site on m-chromosomes (Figure 3 and Figure 4L–T; Table 2). Dual-color FISH analysis reveals that JSJR has 14 5S rDNA sites, ten of which partially overlap with the 45S rDNA sites on the same chromosome regions, and two are located at the interstitial regions of the long arms (L-INT) of a pair of t-chromosomes—similar to the corresponding chromosomes in cytotypes A and B (Figure 3(A1–A4) and Figure 4L). Notably, both 45S and 5S sites coexist at the centromeric regions of the long arms of chromosome pair 3 in JSJR, identical to the corresponding chromosome pairs (chromosome 2 or 4) in cytotype A populations. Notable, one t-chromosome in GXBM (a member of chromosome pair 7) carries an L-PROX-45S rDNA site—a feature not observed in any of the other eight populations. Dual-color FISH shows this chromosome also possesses an L-INT-5S rDNA site, indicating homology with the t-chromosomes bearing L-PROX-(45S + 5S) rDNA site as well as L-INT-5S rDNA site in cytotype A. Additionally, all populations display varying degrees of heterozygosity regarding the presence/absence and size of both DAPI bands and 45S rDNA sites.

### 2.3. Geographic Differentiation Patterns Among the Three Cytotypes

**Figure 8:**
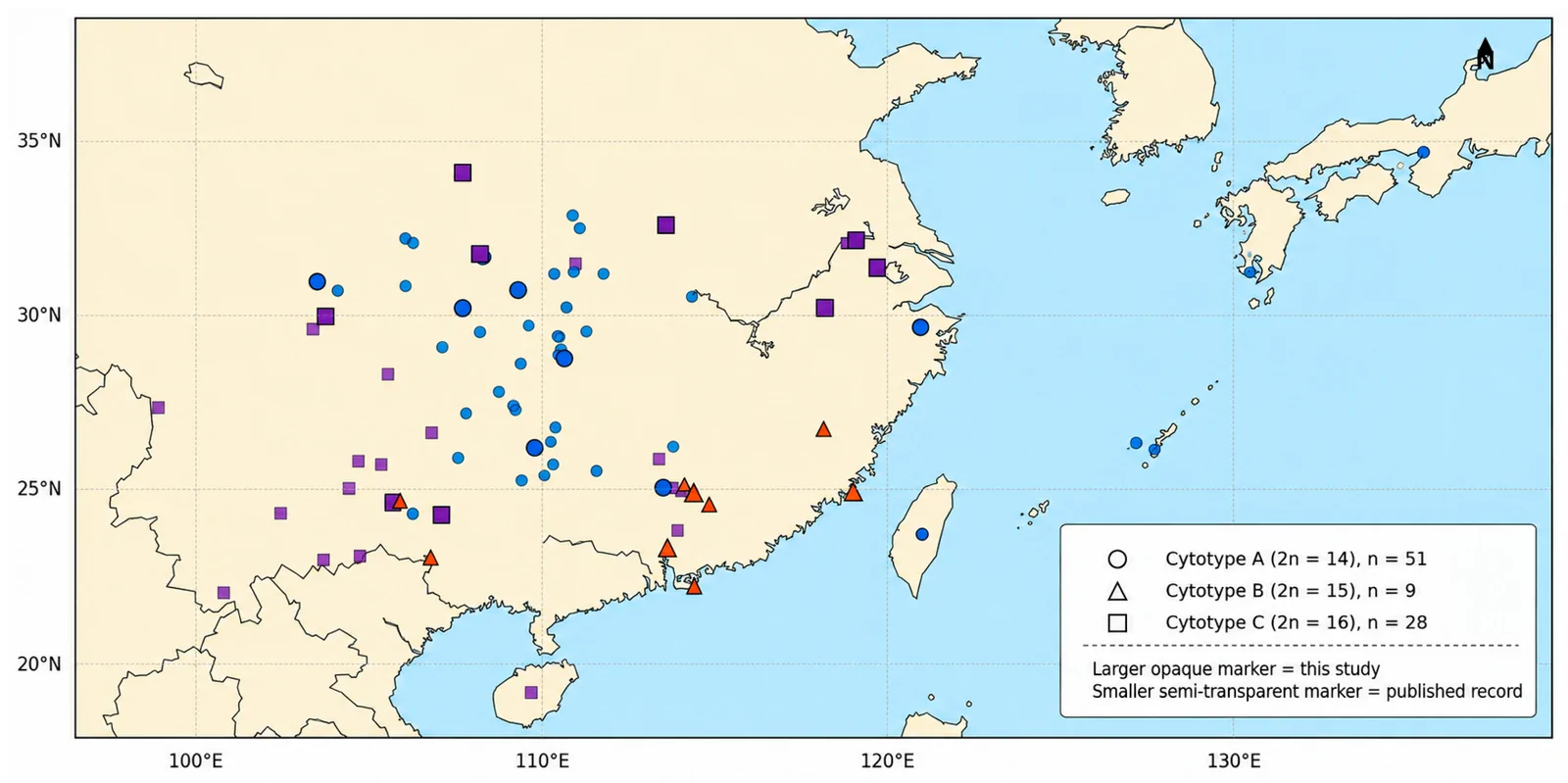
Cytogeographic distribution of the three predominant cytotypes of the *Lycoris aurea* complex. Cytotype A (2*n* = 14), cytotype B (2*n* = 15), and cytotype C (2*n* = 16) are represented by blue circles, red triangles, and purple squares, respectively. Lager opaque markers indicate populations investigated in the present study, whereas smaller semi-transparent markers indicate previously published cytological records. Geographic records and their literature sources are provided in Table S4.

Cytogeographic mapping of 88 documented populations—comprising 51 assigned to cytotype A, 9 to cytotype B, and 28 to cytotype C—revealed a distinct spatial segregation pattern among the three cytotypes within the *L. aurea* complex: cytotype C was predominantly distributed in southwestern and southern China; cytotype A was dominant in central China and the East Asian islands; and cytotype B exhibited a highly restricted distribution centered on the Nanling transitional zone (Figure 8).

Cytotype A possesses a broad distribution range, extending northward to northwestern Hubei, southward to northern Guangxi, and eastward to eastern Zhejiang, Taiwan Island, the Ryukyu Archipelago, and Kagoshima. Its core distribution encompasses the entirety of Hubei and Hunan provinces and extends contiguously into parts of Sichuan Province, Chongqing Municipality, Guizhou Province, and Guangxi Zhuang Autonomous Region—forming a topographically continuous inland mountainous region devoid of major geographical barriers. Cytotype B is restricted to the eastern Nanling transitional zone, and the coastal regions of southeastern South China, exhibiting an extremely narrow and highly fragmented distribution. Cytotype C is widely distributed across the mountainous regions of southwestern and southern China and predominates along the southern and western margins of the species’ overall distribution range. Its range encompasses three primary geographic sectors: (i) southern southwestern China—including the whole territory of Yunnan Province, and parts of Guizhou province, Sichuan province, and Chongqing Municipality; (ii) southern China—covering portions of Guangdong, Guangxi, and Hunan provinces, as well as Hainan Island; and (iii) eastern China—extending to localized areas in Anhui and Jiangsu provinces. It predominates in low-latitude mountainous regions and along the northern margin of the tropical zone, with dense populations concentrated in Yunnan, western Guangxi, and southern Guizhou. Its geographic distribution is disjunct along an east–west axis, comprising two major range segments: the Yunnan–Guizhou Plateau in southwestern China and the Jianghuai region in eastern China; the eastern Nanling Mountains—including Baise City (Guangxi) and Shaoguan City (Guangdong)—constitute a mosaic zone where cytotype C co-occurs with cytotypes A and B. Its range extends westward to the eastern flank of the Hengduan Mountains (e.g., Fugong, Yunnan Province) and northward to the northern foothills of the Qinling Mountains (e.g., Mei County, Shaanxi Province), rendering it the cytotype with the westernmost, southernmost, and northernmost documented distribution limits within the *L. aurea* complex.

## 3. Discussion

### 3.1. Inter- and Intra-Cytotype Karyotypic Differentiation

In this study, molecular cytogenetic karyotyping was conducted on 20 natural populations of the *L. aurea* complex sampled across 13 provinces in China, comprising eight populations of cytotype A (2*n* = 14), three of cytotype B (2*n* = 15), and nine of cytotype C (2*n* = 16). No populations exhibiting 2*n* = 12 or 2*n* = 13 were identified. Consistent with Wang et al.’s research [6], our findings corroborate that these three cytotypes constitute the predominant karyotypic variants within the *L. aurea* complex. Centromere positions were determined using CPD staining and rDNA FISH, and chromosome morphology was classified according to the standardized nomenclature of Levan et al. [47]. A single st-chromosome was observed in four cytotype A populations and one cytotype C population; two st-chromosomes were detected in one cytotype B population. No statistically significant differences in TDL were detected among the three cytotypes—a finding consistent with Shu et al. [7], who reported minimal genome size divergence between 2*n* = 14 and 2*n* = 16 *L. aurea* populations.

Our results demonstrate a consistent, stepwise increase in M_CA_, CV_CL_, and CV_CI_ values from cytotype A to cytotype B and finally to cytotype C—indicating a parallel elevation in intra-chromosomal asymmetry, inter-chromosomal asymmetry, and centromere position heterogeneity, respectively. This represents a canonical pattern in plant karyotype evolution: a progressive increase in overall karyotype asymmetry concomitant with a decreasing proportion of m-chromosomes and a corresponding increase in t/st-chromosomes. The original six-variable PCoA and the UPGMA dendrograms based on six karyotypic parameters both showed pronounced differentiation in overall karyotypic characteristics among the three cytotypes. Cytotype A was clearly separated from cytotypes B and C in multivariate karyotypic space, whereas cytotypes B and C showed greater overall karyotypic similarity to each other. Importantly, this broad ordination pattern was retained in the sensitivity PCoA after exclusion of *x*, 2*n*, and CV_CI_, indicating that the observed differentiation was not solely driven by chromosome-number variables or redundant asymmetry indices. Furthermore, both PCoA, and UPGMA clustering consistently indicated marked differences in the extent of inter-population karyotypic differentiation, particularly between cytotypes A and C: cytotype C displayed high intra-cytotypic karyotypic polymorphism, whereas cytotype A showed high inter-population karyotypic uniformity. Chromosomal heteromorphism—defined as consistent, heritable asymmetry in length and arm ratio between homologous chromosomes—was observed in multiple chromosome pairs across the majority of cytotype A and cytotype C populations. In contrast, the karyotype of cytotype B exhibits pronounced structural divergence, precluding unambiguous homologous chromosome identification based on both morphological criteria and the similarity of DAPI banding and rDNA FISH patterns.

Our fluorochrome banding and rDNA FISH analyses revealed pronounced variation in the number, position and size of both DAPI bands and 45S rDNA sites across the 20 studied populations—indicating extensive intra- and inter-cytotypic polymorphism in AT-rich heterochromatin organization and rDNA array architecture. The distinct DAPI banding and rDNA FISH patterns can serve as robust molecular cytogenetic markers for population discrimination within the *L. aurea* complex. DAPI banding and rDNA FISH patterns are highly conserved across populations of cytotype A, whereas pronounced inter-population variation is evident in cytotype C. This contrast reflects minimal molecular-cytogenetic divergence among cytotype A populations, but marked inter-population differentiation within cytotype C at the molecular-cytogenetic level—a pattern consistent with the differential extent of karyotypic divergence between the two cytotypes, as determined by multivariate analysis of karyotype parameters. Within cytotype B, populations FJHA and GDGZ exhibit nearly identical DAPI banding and rDNA FISH patterns—both of which are markedly distinct from those of JXQN. This molecular-cytogenetic divergence is congruent with the topological relationships among the three populations in the UPGMA dendrogram. Our findings demonstrate that intraspecific dysploidy serves as the primary mechanism driving karyotypic differentiation among cytotypes within the *L. aurea* complex, resulting in systematic variation in chromosome number and pronounced alterations in karyotype asymmetry [6,30,48]. While karyotypic diversification within cytotypes should be driven by multiple interrelated mechanisms: (1) chromosomal structural rearrangements—including pericentric inversions, unbalanced reciprocal translocations, deletions, and duplications—that directly modify chromosome length and arm ratio [24,34,49]; (2) quantitative variation in heterochromatin content, particularly the expansion or contraction of AT- and GC-rich heterochromatic blocks (e.g., 45S rDNA), which alters the cytologically apparent chromosome length [50]; notably, the st-chromosome observed in certain populations arises from localized amplification of 45S rDNA sequences on the short arm, resulting in its conspicuous elongation; (3) geographic isolation, which impedes gene flow and thereby promotes population-level karyotypic differentiation [8,49]; this factor is further discussed in Section 3 of the Discussion with respect to its differential effects on the karyotypic differentiation of the three cytotypes; and (4) the predominant asexual reproductive mode of *L. aurea* promotes the fixation of chromosomal structural variations [37,51]. Cytotype B—a sterile, obligately asexual lineage [6]—lacks any capacity for sexual recombination; consequently, chromosomal structural variants accumulate and become fixed more readily in this cytotype than in cytotypes A and C, which maintain facultative sexual–asexual reproductive strategies. This differential mode of inheritance underlies the heightened chromosomal heteromorphism observed in cytotype B.

The variation in DAPI band and rDNA site—both critical features of karyotypic differentiation in the *L. aurea* complex—arises from the dynamic evolutionary processes acting upon specific repetitive DNA sequences. DAPI bands almost exclusively correspond to constitutive heterochromatin, specifically AT-rich tandemly repeated satellite DNA arrays [52]. Consequently, interspecific and intraspecific variation in DAPI banding pattern serves as a cytological proxy for satellite DNA evolution, which is driven by molecular drive mechanisms such as unequal crossing over, transposition, rolling-circle replication, and replication slippage. These mechanisms collectively generate novel sequence variants, modulate copy number, and reshape nucleotide composition—thereby yielding extensive polymorphism across populations in the presence, relative abundance, and chromosomal distribution of DAPI-positive heterochromatic regions [53,54]. Similarly, the extensive interspecific and intraspecific variability observed in plant rDNA arrays stems from the inherent instability of tandemly repeated sequences, compounded by highly active recombination and transposition mechanisms operating under weak selective constraints [55]. The tandem organization of rDNA arrays predisposes homologous chromosomes to misalignment and then unequal crossing over during meiosis; moreover, the high sequence conservation among rDNA repeats promotes ectopic recombination between non-allelic loci—particularly within subtelomeric and telomeric chromosomal regions [55,56]. Such recombination events—including unequal crossing over and ectopic recombination—can result in the complete elimination of rDNA arrays, accounting for the absence of 45S rDNA signals on the short arms of one to three t chromosomes in ZJSZ, FJHA, GZGD, and JXQN populations. Copy number variation arising from unequal crossing over further underlies the size dimorphism of homologous 45S rDNA loci on short arms of t-chromosome observed in all the populations studied. In addition, ectopic recombination can shift rDNA sites to novel chromosomal positions or facilitate the emergence of entirely new sites; retrotransposition—predominantly involving 5S rDNA—and the re-integration of extrachromosomal circular rDNA (ecc rDNA) represent additional molecular pathways capable of generating de novo rDNA insertions [55].

### 3.2. Evolutionary Relationships Among the Three Cytotypes

The higher inter-population karyotypic polymorphism observed in cytotype C—relative to cytotype A—is broadly consistent with previously reported chloroplast DNA data, which show that the 2*n* = 16 cytotype exhibits the highest nucleotide diversity among the four recognized cytotypes [6]. This pattern suggests a longer and/or more complex evolutionary history of differentiation within cytotype C, leading to the accumulation of a greater number of lineage-specific mutations [57]. In contrast, cytotype A displays the highest degree of karyotypic uniformity across the three cytotypes. Collectively, these convergent lines of evidence support cytotype C (2*n* = 16) as the putative ancestral karyotype of the *L. aurea* complex, whereas cytotype A appears to represent a putatively derived cytotype.

The eight cytotype A populations consistently exhibit two diagnostic molecular cytogenetic features: (i) one pair of m-chromosomes bearing CEN- or S/L-PCEN-(45S + 5S) rDNA sites, and (ii) one pair of t-chromosomes harboring both L-INT-5S rDNA site and L-PROX-(45S + 5S) rDNA site. These same features are present in five previously reported 2*n* = 14 populations—HNYL (Yuanling, Hunan), HBFX (Fangxian, Hubei), HBBD (Badong, Hubei), GZDS (Dushan, Guizhou), and HNXN (Xinning, Hunan) [11,18]. The mitotic chromosome preparations for the HNYL population reported by Quan et al. were obtained from root tips of a bulb distinct from that sampled in the present study—thereby providing independent confirmation of intra-population uniformity for these cytogenetic markers [18]. These 12 cytotype A populations analyzed by our group using rDNA FISH were sampled across the vast majority of this cytotype’s documented geographical distribution on the Chinese mainland, with the exception of Guangxi Zhuang Autonomous Region. The foregoing evidence suggests that all 2*n* = 14 populations of the *L. aurea* complex on the Chinese mainland share a recent common ancestry, tracing back to a single ancestral lineage. The preceding analysis, based on comparative patterns of karyotypic differentiation among cytotypes, suggests that cytotype C predates cytotype A in the evolutionary history of the *L. aurea* complex. Indeed, comparative analysis of rDNA FISH patterns provides independent support for this inference. If cytotype A had preceded cytotype C in evolutionary origin and cytotype C subsequently arose from cytotype A via centric fission, then a majority—or at least a substantial proportion—of cytotype C populations would be expected to retain a pair of t-chromosomes bearing the L-PROX-(45S + 5S) rDNA sites. However, none of the nine cytotype C populations newly examined in this study, nor the three previously reported populations (HBWF from Wufeng, Hubei; GXTL from Tianlin, Guangxi; HNZX from Zixing, Hunan) [11], exhibit this pattern. Critically, only GXBM carries a single t-chromosome with the L-PROX-(45S + 5S) rDNA site, thereby failing to provide empirical support for the centric fission hypothesis.

**Figure 9:**
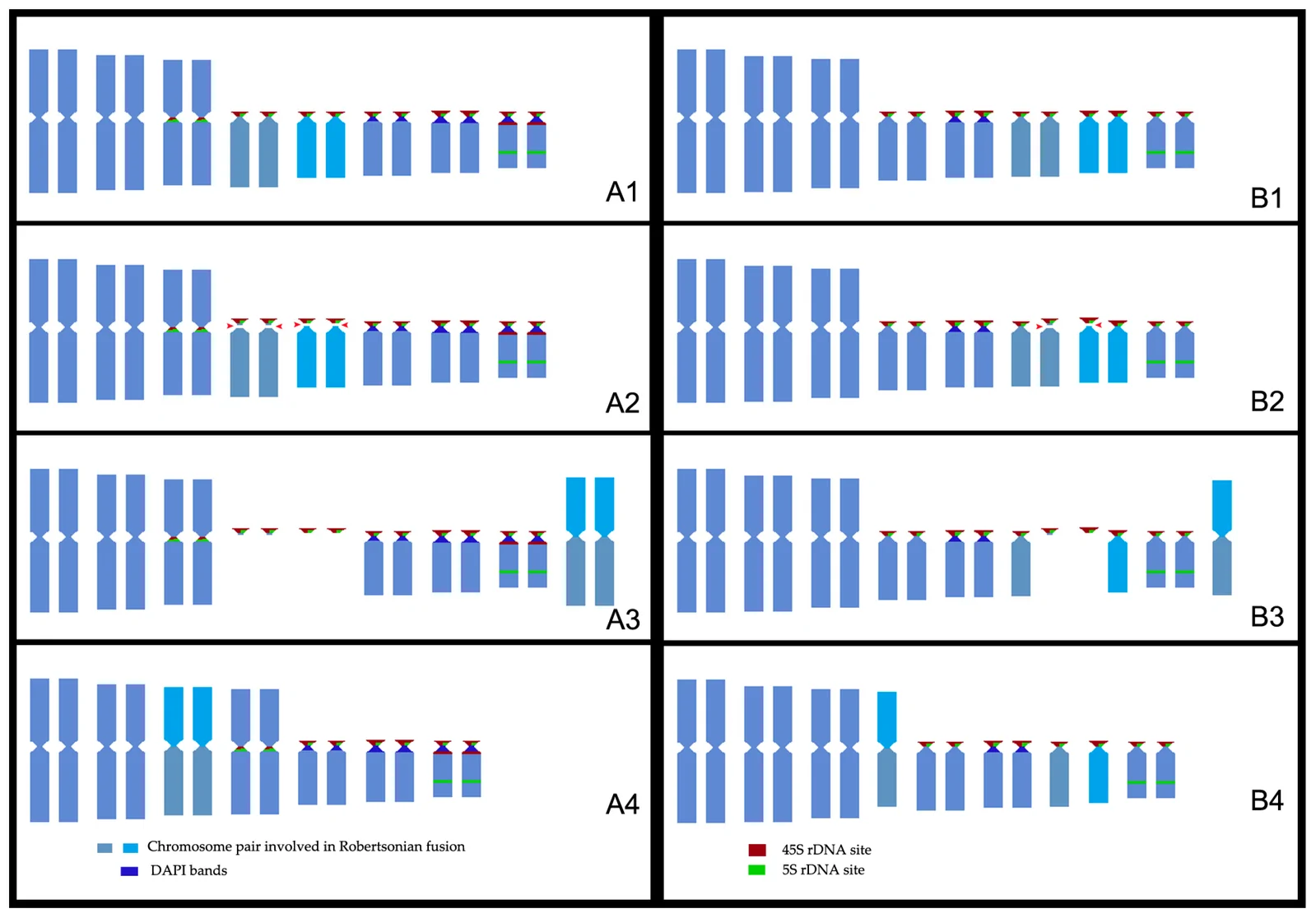
Schematic representation of the working hypothesis proposing that cytotypes A and B arose independently from distinct populations of cytotype C via two and one Robertsonian fusion events, respectively. (**A1**,**B1**) the putative ancestral karyotypes of cytotypes A and B, respectively, both with a karyotype of 2*n* = 16 = 6m + 10t (cytotype C), but differing in DAPI banding and rDNA FISH patterns. The putative ancestral karyotype of cytotype A should exhibit three defining features: (i) at least three pairs of t-chromosomes displaying (CEN + L-PCEN)-DAPI bands—exemplified here by three such t-chromosome pairs; (ii) one pair of m-chromosomes harboring a CEN- or S/L-PCEN-(45S + 5S) rDNA site; and (iii) one pair of t-chromosomes bearing an L-PROX-(45S + 5S) rDNA site. The putative ancestral karyotype of cytotype B is inferred to contain one or two pairs of t-chromosomes exhibiting (CEN + L-PCEN)-DAPI bands—represented here by one such pair—and to lack t-chromosomes bearing the L-PROX-(45S + 5S) rDNA site. Moreover, the short arms of all five pairs of t-chromosomes in the putative ancestral karyotypes of cytotype A and B each harbor a 45S rDNA site. (**A2**,**B2**) Chromosomes undergoing Robertsonian translocation experience centromeric breakage (red arrowheads indicate the breakpoints), and the translocations involve two pairs of t-chromosomes. Breakpoints may also occur in the pericentromeric regions. (**A3**,**B3**) The long arms of the broken t-chromosomes fuse to form two (a pair) or one m-chromosomes, respectively. (**A4**,**B4**) The short arms of the broken t-chromosomes are eliminated, resulting in the derived karyotypes of cytotype A (2*n* = 14 = 8m + 6t) and cytotype B (2*n* = 15 = 7m + 8t), respectively. If the t-chromosomes participating in a Robertsonian fusion bearing (CEN + L-PCEN)-DAPI bands, the resulting m-chromosome will harbor an S/L-PCEN-DAPI bands. When one t-chromosome exhibits a breakpoint in the pericentromeric region of the short arm and the other in the pericentromeric region of the long arm, the resulting m-chromosome carries a S/L-PCEN-45S rDNA site. To clearly illustrate homology, centromere position, and fusion points, chromosomes participating in Robertsonian translocations are depicted in distinct colors—different from those used for non-Robertsonian chromosomes—and the two chromosome pairs involved are further differentiated using unique color assignments.

The origin of cytotype B (2*n* = 15) is constrained to three mutually exclusive evolutionary scenarios, as proposed by Kurita [16]. Our rDNA FISH analysis revealed that none of the three cytotype B populations examined possess t-chromosomes bearing the L-PROX-(45S + 5S) rDNA sites. This finding makes two alternative evolutionary origins of the 2*n* = 15 karyotype less plausible: (i) centric fission of an m-chromosome in cytotype A—which would be expected to produce progeny retaining two t-chromosomes each carrying the L-PROX-(45S + 5S) rDNA site; and (ii) intraspecific hybridization between cytotype A and cytotype C—which would be expected to yield progeny possessing at least one t-chromosome bearing this rDNA site pattern. In contrast, the molecular-cytogenetic data are most parsimoniously explained by a single Robertsonian translocation event between two t-chromosomes in a putative ancestral population with a karyotype of 2*n* = 16 = 6 m + 10 t. This ancestral karyotype would be characterized by the presence of only one or two pairs of telocentric chromosomes displaying (CEN + L-PCEN)-DAPI bands and—critically—the absence of the t-chromosome bearing the L-PROX-(45S + 5S) rDNA site. Such a rearrangement yields the derived cytotype B (2*n* = 15 = 7m + 8t), as illustrated in Figure 9(B1–B4).

The current molecular-cytogenetic evidence further refutes the hypothesis that cytotype A arose from cytotype B via a Robertsonian fusion event involving two t-chromosomes. Cytotype B lacks two molecular-cytogenetic hallmarks consistently observed in cytotype A: (i) no t-chromosomes bear the L-PROX-(45S + 5S) rDNA site; and (ii) only one or two t-chromosome pairs exhibit (CEN + L-PCEN)-DAPI bands—whereas in cytotype A, nearly all t-chromosomes display these bands, with the single exception of HBES, which lacks the DAPI signal on one t-chromosome. Collectively, these observations—combined with the prior inference that cytotype C is the putative ancestral karyotype—favor an alternative hypothesis: cytotype A originated independently from a distinct 2*n* = 16 ancestral population through two simultaneous Robertsonian translocations involving four t-chromosomes (Figure 9(A1–A4)). Under this working hypothesis, the putative ancestral cytotype C population that gave rise to cytotype A is predicted to exhibit three molecular-cytogenetic features, consistent with the conserved molecular-cytogenetic signatures observed across all cytotype A populations: (i) at least six t-chromosomes bearing (CEN + L-PCEN)-DAPI bands; (ii) one pair of m-chromosomes harboring CEN- or S/L-PCEN-(45S + 5S) rDNA sites; and (iii) one pair of t-chromosomes carrying the L-PROX-(45S + 5S) rDNA sites. Among the nine cytotype C populations analyzed in this study, GXBM, GXTL, and SCEMS satisfy criterion (i); JSJR satisfies criterion (ii); however, no population fulfills all three criteria simultaneously. Notably, a single t-chromosome bearing the L-PROX-(45S + 5S) site was detected in GXBM—providing direct evidence that this rDNA site is present, albeit rarely, within cytotype C. The failure to identify a cytotype C population that fully satisfies all three inferred ancestral karyotypic criteria is likely attributable to two non-exclusive factors: first, the limited geographic and taxonomic sampling—particularly the absence of cytotype C samples from Yunnan, Guizhou, and Guangdong provinces in our FISH analyses; and second, the possibility that the actual cytotype C progenitor of cytotype A has subsequently diverged and lost one or more of these diagnostic features. Resolving this ambiguity requires expanded sampling across the full distribution range of cytotype C, followed by integrated molecular cytogenetic analyses.

In summary, the current cytogenetic evidence is consistent with a working hypothesis in which cytotype C (2*n* = 16) represents the ancestral karyotype of the *L. aurea* complex, whereas cytotypes A (2*n* = 14) and B (2*n* = 15) likely originated independently via two and one Robertsonian fusion events, respectively (Figure 9). Under this scenario, the evolutionary pathways C → A and C → B offer a parsimonious explanation for the observed differences in chromosome number, karyotypic morphology, as well as DAPI banding and rDNA FISH patterns. However, none of the cytotype C populations examined in this study retains the complete set of molecular-cytogenetic features inferred to be ancestral, and geographic sampling remains incomplete across all three cytotypes. Consequently, these inferred evolutionary pathways should be regarded as cytogenetically informed hypotheses—not definitive reconstructions—of historical divergence events. This evolutionary hypothesis necessitates comprehensive geographic sampling across the full distribution range of the species complex, coupled with rigorous empirical validation through integrated molecular cytogenetic and molecular phylogenomic approaches. Specifically, FISH, genomic in situ hybridization (GISH), and chromosome painting should be applied to resolve centromere architecture and precisely map chromosomal fusion breakpoints. Concurrently, whole-genome sequencing should be performed, and a high-resolution phylogenomic framework—reconstructed from concatenated nuclear and chloroplast genomic loci—should be employed to jointly infer lineage divergence history and the temporal sequence of chromosomal rearrangements.

Dysploidy is a major evolutionary mechanism underlying both speciation and karyotypic diversification in angiosperms and ferns, generating intraspecific or intrageneric variation in basic chromosome number [30,49]. Ascending dysploidy—characterized by an increase in chromosome number—is primarily driven by centric fission; descending dysploidy—marked by chromosome number reduction—arises predominantly through three types of centric fusion: Robertsonian translocations, end-to-end translocations, and nested chromosomal insertions [30]. Empirical evidence indicates that centric fusion occurs significantly more frequently than centric fission in plants [30,48]. This asymmetry stems from fundamental mechanistic constraints: centric fission requires several sequential, low-probability molecular and cytological events to yield stably inherited derivatives—including de novo telomere addition at broken chromosome ends, reconstruction of dual functional kinetochores, faithful trivalent segregation during meiosis I, production of balanced euploid gametes, and population-level fixation of the homozygous state. In contrast, centric fusion—particularly Robertsonian translocation—readily achieves meiotic stability without requiring de novo telomere synthesis or kinetochore reorganization, incurs minimal fertility costs, and may be favored by centromeric drive, thereby facilitating diploidization [30,58]. Notably, although Robertsonian translocations dominate karyotype evolution in mammals, they remain comparatively rare in flowering plants [48].

The dysploidy in *Lycoris* has been extensively documented and remains a subject of ongoing scholarly debate. Inariyama advanced a Robertsonian fusion hypothesis to account for karyotypic evolution within the genus, proposing that all fertile diploid karyotypes in *Lycoris* originated from the ancestral 2*n* = 22 karyotype of *L. sanguinea*—characterized exclusively by acrocentric (I-shaped) chromosomes—through successive centric fusions, resulting in progressively reduced chromosome numbers [20,31]. Under this model, the highly derived karyotype of *L. aurea* (2*n* = 12 = 10m + 2t) is interpreted as the outcome of repeated fusion of acrocentric chromosome pairs, thereby supporting x = 11—not x = 6—as the ancestral basic chromosome number for this genus. In contrast, Flory proposed an alternative centric fission hypothesis, suggesting that acrocentric I-shaped chromosomes originate from the fission through the centromeric region of metacentric (V-shaped) chromosomes [32]. Kurita proposed that the centric fission hypothesis is the most probable mechanism for the karyotype evolution of *Lycoris*, and that the karyotype comprising of M and T chromosomes may be ancestral [10], based on facts such as the DNA content of genomes composed of M and T being larger than that of genomes composed solely of A chromosomes [59]. Chang et al. proposed that T chromosomes arise from centromeric fission of M chromosomes, as FISH detected a large block of 45S rDNA and 5S rDNA adjacent to telomeric repeats at the physical ends, and the presence of abundant repetitive sequences suggests that these repeats are amplified to seal the broken ends [33]. Recently, Zhang et al. proposed that *L. aurea* (2*n* = 14 = 8m + 6t) represents the plesiomorphic state within the genus *Lycoris*, on the basis of karyotypic asymmetry indices and Stebbins’ widely accepted hypothesis that plant karyotype evolution proceeds from symmetry toward increasing asymmetry [26]. However, accumulating cytogenetic and phylogenetic evidence from multiple independent studies supports Robertsonian fusion as the primary mechanism of karyotypic evolution in *Lycoris*. Liu et al. employed FISH with rDNAs and centromeric histone H3 variant associated DNAs as probes to identify multiple chromosomal structural rearrangements in *Lycoris*, specifically centric fusion, pericentric inversion, ribosomal gene amplification, and segmental deletion [34]. In a detailed cytogeographic study of *L. radiata*, Liu et al. demonstrated that metacentric (m) and small metacentric (m’) chromosomes arose through Robertsonian fusion of two acrocentric progenitors in geographically localized populations [25]. The comprehensive cytogeographic analysis of the *L. aurea* complex conducted by Wang et al. revealed that the cytotype 2*n* = 12 is restricted to a narrow, localized area, whereas the cytotypes 2*n* = 14 and 2*n* = 16 exhibit broad geographic distributions [6]. Based on their extensive distribution and high frequency in surveyed populations, the latter two cytotypes are inferred to represent the ancestral karyotypic lineage within the species [6]. Furthermore, multiple independent molecular phylogenetic analyses consistently place species harboring the putatively ancestral all-acrocentric karyotype (2*n* = 22A)—including *L. haywardii*, *L. sprengeri*, and *L. radiata* var. *pumila*—at basal positions within the *Lycoris* phylogeny, supporting their early divergence in the evolutionary radiation of this genus [6,60,61,62]. Critically, our comparative molecular cytogenetic analysis of the three major cytotypes in the *L. aurea* complex provides robust cytogenetic evidence consistent with the working hypothesis that intraspecific variation in chromosome number arises through descending dysploidy driven by recurrent Robertsonian fusions.

### 3.3. Biogeographic Drivers of Intraspecific Karyotypic Differentiation

Cytogeographic analyses—grounded in comparative assessment of chromosomal variation across geographically structured populations—facilitate inference of putative centers of origin, divergence chronologies, and historical range expansion routes [6,25,63]. Such integrative studies clarify how ploidy shifts and structural chromosomal rearrangements contribute to ecological niche differentiation and biogeographic patterning in plants, thereby providing mechanistic insights into present-day distributional limits and range disjunctions [25]. When synthesized with paleogeographic reconstructions and paleoclimatic data, cytogeographic patterns enable a temporally calibrated, spatially explicit reconstruction of species evolutionary history—including its tempo, mode of range dynamics, abiotic and biotic drivers, and lineage-specific genomic responses [8].

Paleoclimatic ecological niche modeling (ENM) of the East Asian *L. aurea* complex, as conducted by Meng et al. yielded four key findings [8]: (1) Precipitation predominantly shapes the distribution of cytotype A (2*n* = 14), whereas temperature serve as the primary climatic determinant for cytotype C (2*n* = 16), indicating that the two cytotypes exhibit different climatic sensitivities. (2) Temporal analysis of habitat suitability indicates a pronounced contraction during the Last Interglacial (LIG), confining suitable habitats to refugia located in southwestern and southern China, Taiwan Island, and the Ryukyu Islands. A subsequent range expansion took place during the Last Glacial Maximum (LGM), characterized by westward and northward shifts of continental populations. The current distribution pattern was largely established by the Mid-Holocene. (3) Three historically significant migration corridors were identified: (i) the Central Sichuan–Chongqing–Hubei corridor, characterized by high gene flow and sympatric distribution of cytotypes A and C; (ii) the Yunnan Plateau corridor, where dispersal is restricted exclusively to cytotype C; and (iii) the Nanling Mountains corridor—situated at the tri-provincial junction of Guangdong, Guangxi, and Hunan—where cytotypes A, B and C co-occur and localized, short-distance genetic differentiation is ongoing. (4) Taiwan Island and the Ryukyu Archipelago functioned as stable, long-term insular refugia exclusively inhabited by cytotype A. During glacial periods, the exposed East China Sea continental shelf constituted a filter corridor dominated by arid, open-habitat conditions—effectively restricting the dispersal of cytotypes B and C to these offshore islands. Integrating these findings with the working hypothesis—derived from our molecular-cytogenetic evidence—regarding the karyotypic evolutionary pathways of the three predominant cytotypes (C → A and C → B), together with the geographic patterns of cytotype differentiation uncovered in this study, yields crucial insights into the spatiotemporal dynamics of cytotype evolution within the *L. aurea* complex and the multilevel drivers shaping its present-day distribution.

Paleoclimatic ENM of the *L. aurea* complex revealed glacial refugia in southwestern China [8]. Cytogeographic analysis further indicates that cytotype C (2*n* = 16) is predominately distributed in the subtropical mountainous regions of southwestern and southern China. Integrating these findings with the hypothesis—based on molecular cytogenetic evidence—that cytotype C represents the ancestral karyotype, we propose that the Yunnan-Guizhou Plateau and adjacent regions of southern China may constitute the primary center of origin and early diversification for this species complex. Cytotype C may have experienced prolonged independent evolution, driven by extreme topographic fragmentation and persistent geographic isolation across its range—factors that severely constrained interpopulation gene flow. Consequently, disjunct populations may have accumulated lineage-specific microstructural chromosomal variants, including positional shifts and copy-number variations in rDNA sites, gains or losses of DAPI-positive heterochromatic blocks, and minor chromosomal rearrangements. Moreover, pronounced habitat heterogeneity across microhabitats may have imposed divergent selective pressures, thereby accelerating karyotypic differentiation among geographically isolated populations. During the LIG, suitable habitats for *L. aurea* underwent severe contraction, confining cytotype C to low-latitude refugia in Southwest and South China—regions where prolonged isolation may have fostered the accumulation of extensive karyotypic polymorphism [8]. Subsequent cooling during the LGM expanded overall habitat suitability [64], and may have triggered northward range expansion of cytotype C populations along the three major corridors [8]. We hypothesize that a cytotype C population possessing distinct cytogenetic features underwent two concurrent Robertsonian fusions within the Sichuan–Chongqing–Hubei corridor, giving rise to cytotype A; this derived 2*n* = 14 cytotype subsequently underwent rapid eastward and northward dispersal along the topographically continuous Wuling–Yangtze mid-mountain corridor, ultimately colonizing Taiwan Island and the Ryukyu Archipelago. In contrast to cytotype C, uninterrupted gene flow across the topographically contiguous hills and basins of Central China may have constrained the emergence of novel karyotypic variation, thereby maintaining high karyotypic uniformity across cytotype A populations. Likewise, geographically isolated cytotype C populations in the eastern Nanling refugia likely experienced a single Robertsonian fusion event, leading to the origin of cytotype B (2*n* = 15). East–west-oriented topographic barriers associated with the Nanling Mountains and fragmented coastal hills may impede large-scale range expansion of cytotype B, which remains restricted to the transitional ecotone between cytotypes A and C. The Yunnan Plateau corridor may permit only intra-cytotype dispersal of cytotype C [8]. During the LGM, the arid, exposed continental shelf of the East China Sea functioned as a dispersal filter [64], allowing colonization of Taiwan Island and the Ryukyu Archipelago exclusively by the precipitation-sensitive cytotype A, whereas temperature-sensitive cytotype C may have been excluded [8]. Postglacial sea-level rise submerged the East China Sea land bridge, establishing permanent oceanic vicariance. Following colonization, insular cytotype A populations may have experienced independent karyotypic differentiation, resulting in the rDNA FISH patterns that are distinct from those observed in their continental conspecifics [33]. Furthermore, recurrent secondary Robertsonian fusions in island lineages may have occurred, yielding rare karyomorphs with chromosome numbers of 2*n* = 13 and 2*n* = 12 [9,10].

Persistent climatic niche filtering may have driven the parapatric spatial segregation of the two dominant cytotypes, A and C [8]. The distribution of cytotype A is primarily constrained by precipitation-related climatic variables [8], likely enhancing its fitness in temperate inland mountain regions of central China—areas characterized by pronounced seasonal variability in both temperature and moisture. In contrast, cytotype C is primarily governed by temperature-related climatic variables [8], which likely enables it to prevail in warm, humid karst landscapes and high-elevation plateaus across southwestern and southern China. Although their fundamental climatic niches exhibit substantial overlap [8], divergent sensitivities to thermal and hydrological gradients have likely driven pronounced spatial partitioning: cytotype A predominates in central and northern subtropical continental regions; cytotype C prevails in low-elevation areas of southern and southwestern China; and the Nanling Mountains form a broad ecotonal transition zone that supports cytotypes A, B and C.

In summary, intraspecific karyotypic differentiation in the *L. aurea* complex may have arisen from the synergistic interplay between intrinsic chromosomal structural variation—specifically, independent Robertsonian fusion events occurring in geographically isolated subpopulations of the putative ancestral cytotype (2*n* = 16)—and extrinsic drivers including Quaternary climatic oscillations, topographic barriers, and ecological niche divergence. Prolonged climatic niche filtering may have stabilized the contemporary cytogeographic distribution pattern. It is important to emphasize that the biogeographic and evolutionary scenarios proposed herein for the three cytotypes of the *L. aurea* complex represent a hypothesis-generating, integrative synthesis—not a direct historical reconstruction. The current cytogeographic dataset integrates contemporary sampling with published locality records but lacks temporal calibration for chromosomal rearrangements, refugial persistence, range shifts, or population divergence. Consequently, the spatial congruence among cytotype distributions, previously published paleoclimatic niche reconstructions, and karyotypic evolutionary pathways is consistent with—yet insufficient to confirm—a Pleistocene scenario involving refugial isolation, postglacial range expansion, and subsequent karyotypic differentiation. Rigorous testing of these hypotheses requires denser geographic sampling, and integrated analyses using molecular cytogenetics, molecular phylogenomics, population genomics, and phylogeography.

## 4. Materials and Methods

### 4.1. Plant Materials

Twenty natural populations of the *L. aurea* complex were collected from 20 counties across 13 provinces in China (Table 1) and maintained under standardized ex situ conditions in the Germplasm Resource Garden of Huaihua University. To avoid biasing sampling, bulbs of the same population were collected apart from more than five meters each other. Detailed collection information—including the administrative divisions of collection location, and precise geographical coordinates—is provided in Table S1. Population identifiers were systematically assigned using a four (five)-character alphanumeric code: the first two characters represent the provincial abbreviation (e.g., “HN” for Hunan Province), and the final two (three) characters denote the county-level administrative unit (e.g., “YL” for Yuanling County).

### 4.2. Chromosome Preparation

For each population, three to five bulbs were employed for root induction and mitotic chromosome preparation. The bulbs were carefully excavated from the soil, transferred to conical flasks for hydroponic root induction, and incubated under controlled conditions. Upon emergence of adventitious roots reaching approximately 2.0 cm in length, excise ~0.3 cm root tips. Rinse thoroughly with double-distilled water (ddH_2_O), followed by treatment with a saturated aqueous solution of α-bromonaphthalene at 28 °C for 3.5 h. Subsequently, fix the samples overnight at 4 °C in freshly prepared fixative composed of methanol and glacial acetic acid (3:1, *v*/*v*). Chromosome slide preparation followed the enzymatic cell wall digestion and flame-drying protocol described by She et al. [36]. The slides exhibiting high mitotic index and well-spread metaphase chromosomes were selected using an Olympus BX51 (Olympus Corporation, Tokyo, Japan) phase-contrast microscope and stored at −20 °C until use.

### 4.3. CPD Staining

Of the 20 populations used in this study, CPD staining was performed sequentially with 45S rDNA FISH on 14 populations, and CPD staining was conducted following dual-color FISH with 5S and 45S rDNA probes on three additional populations. In the former experiment, the CPD staining was carried out in accordance with the protocol described by She et al. [39]. Briefly, chromosome spreads were first incubated with RNase A (100 µg·mL^−1^ in 2 × SSC, 37 °C, 1 h), followed by pepsin digestion (50 µg·mL^−1^ in 0.01 mol L^−1^HCl, 37 °C, 10 min). Subsequently, slides were stained with a fluorochrome mixture containing PI (0.6 µg·mL^−1^) and DAPI (3 µg·mL^−1^) dissolved in 30% (*v*/*v*) Vectashield H100 mounting medium (Vector Laboratories, Burlingame, CA, USA) for about 45 min at room temperature in the dark. The CPD staining following dual-color FISH is conducted as follows: following removal of the coverslip from the hybridized slide, the slide was washed twice with 2 × SSC buffer (10 min per wash). Subsequently, the slide was subjected to graded ethanol dehydration (70%, 90%, and 100%, each pre-chilled to −20 °C), with a 5-min incubation at each concentration. The slide is air-dried thoroughly and then stained with the aforementioned PI and DAPI mixture under the same conditions. Fluorescence imaging was performed using an Olympus BX60 epifluorescence microscope equipped with appropriate UV (for DAPI) and green (for PI) excitation/emission filter sets (Olympus Corporation, Tokyo, Japan). Digital images were acquired using a QImaging Retiga R6 CCD camera controlled by Ocular imaging software 2.0 (Teledyne Photometrics, Tucson, AZ, USA).

### 4.4. DNA Probes and FISH

The 45S rDNA probe for mono-color FISH—applied across 14 populations—was a 9.04-kb genomic fragment isolated from *Solanum lycopersicum* [39], labeled with biotin-16-dUTP via nick translation using a commercial nick translation kit (Roche Diagnostics, Mannheim, Germany). The dual-color FISH probes targeting both 5S and 45S rDNA repeats were the oligonucleotide probes developed by Han et al. [65], and were applied to the metaphase chromosomes from 6 populations. The 5S rDNA probes, designated as 5S-1 and 5S-2, along with the 45S rDNA probes, designated as 45S-1, 45S-2 and 45S-3, were synthesized by Sangon Biotech (Shanghai) Co., Ltd. (Shanghai, China). 5S-1 and 5S-2 were labeled at the 5′-terminus with 6-carboxyl fluorescein (6-FAM) and mixed together to make the 5S rDNA probe solution. 45S-1, 45S-2, and 45S-3 were labeled at the 5′and 3′-termini with 6-carboxyl-tetramethyl rhodamine (TAMRA) and mixed together to make the 45S rDNA probe solution. The full nucleotide sequences of the oligonucleotide probes are presented in Table S3.

FISH with the 45S rDNA probe was performed on slides previously stained with CPD. The slides were washed twice with 2 × SSC buffer for 15 min each at room temperature under lighting condition, dehydrated through a cold graded ethanol series (70%, 90%, and 100%; each pre-chilled to −20 °C), and air-dried at room temperature. In situ hybridization was carried out following the protocol described by She et al. [39]. Biotin-labeled probes were detected using fluorescein-conjugated avidin D (Vector Laboratories, Burlingame, CA, USA).

FISH using both 5S and 45S rDNA oligonucleotide probes was performed according to the protocol described by Wei et al. [37]. Briefly, chromosome slides were baked at 65 °C for 45 min, allowed to cool to room temperature, and then denatured in 70% deionized formamide at 85 °C for 2.5 min. Subsequently, slides were immediately dehydrated through a cold ethanol series (70%, 90%, and 100%, each pre-chilled to −20 °C), followed by air drying in the dark. Hybridization mixture was applied to each slide, which was then sealed and incubated overnight at 37 °C in a humidified chamber. Post-hybridization, slides were washed twice with 2 × SSC buffer at room temperature (5 min per wash), and air-dried in the dark.

Following hybridization and post-hybridization washes, chromosomes were counterstained with 3 µg mL^−1^ DAPI dissolved in Vectashield H-1000 antifade mounting medium, diluted to 30% (*v*/*v*) in double distilled water. Specimens were then coverslipped and examined under the epifluorescence microscope specified above. Digital images were acquired using a QImaging Retiga R6 CCD camera operated through Ocular imaging software 2.0. Fluorescence detection was performed using UV, blue, and green excitation/emission filters for DAPI, Fluorescein/6-FAM, and TAMRA, respectively. Raw images were processed using Adobe Photoshop CS8.0.1 (Adobe Systems Inc., San Jose, CA, USA).

### 4.5. Karyomorphological Analysis

Karyotype analysis was performed according to the standardized protocol outlined by She et al. [40]. For each population, the chromosome number, chromosome morphology, fluorochrome banding patterns, and rDNA FISH signals were systematically verified across multiple metaphase cells derived from independently sampled bulbs (at least five metaphase cells per bulb). Upon confirming intra-population consistency in these core cytogenetic features, a single high-quality metaphase spread—characterized by minimal chromosome overlap, complete chromosomal condensation, well-defined primary constrictions, and unambiguous fluorescence signals—was selected for standardized morphometric analysis using Adobe Photoshop CS3 (version 8.0.1). We acknowledge that morphometric parameters derived from a single representative metaphase spread per population do not capture the extent of intra-populational cell-to-cell variation and should therefore be interpreted primarily as population-level descriptive measurements.

The following linear measurements were obtained for each chromosome: long arm length (L), short arm length (S), fluorescent band (or FISH signal) length, and the distance from the centromere to the center of FISH signal that is located in the proximal or interstitial chromosomal regions. Based on these measurements, the following karyotypic parameters were computed (calculating metrics separately for individual chromosome instead of each chromosome pair): (i) relative chromosome length (RL), expressed as a percentage of TDL; (ii) arm ratio (AR = L/S); (iii) TDL; (iv) proportional representation of each fluorescent band within the diploid complement, expressed as a percentage of the TDL; (v) mean centromeric index (CI) [41]; (vi) four quantitative indices of karyotype asymmetry—CV_CI_ [41], CV_CL_ [41], M_CA_ [42], and Stebbins’ asymmetry category [66]; and (vii) the percent distance from centromere to DNA site (*di* = *d* × 100/*a*; *d* = the distance of the center of the FISH signal to the centromere, *a* = the length of the corresponding chromosome arm). Chromosomes were classified into four structural types—metacentric (m; AR = 1.00–1.70), submetacentric (sm; AR = 1.71–3.00), subtelocentric (st; AR = 3.01–7.00), and telocentric (t; AR > 7.00)—following the nomenclature system proposed by Levan et al. [47]. Chromosome numbering and arrangement in karyotype are standardized based on descending order of chromosome length: for populations with 2*n* = 14 or 2*n* = 16, homologous chromosome pairs are ordered and numbered from longest to shortest; in contrast, for populations with 2*n* = 15, individual chromosomes—rather than pairs—are arranged and numbered sequentially by decreasing length. Ideograms are drawn using quantitative morphometric data combined with position and length information of fluorescent bands and rDNA FISH signals. All 2*n* chromosomes are displayed in the ideograms to enable direct comparison of homologous chromosomes with respect to morphology, as well as fluorochrome banding and rDNA FISH patterns—thereby facilitating the identification of structural heterozygosity and chromosomal rearrangements.

To visualize karyotype asymmetry patterns across the analyzed populations, a bidimensional scatter plot of M_CA_ versus CV_CL_ was generated. To elucidate karyological relationships among the 20 populations, principal coordinate analysis (PCoA) was conducted using Gower’s similarity coefficient, computed from six quantitative karyotypic parameters—*x*, 2*n*, TDL, CV_CI_, M_CA_ and CV_CL_—following the methodology outlined by Peruzzi and Altınordu [43]. A character matrix was constructed based on the six karyotypic parameters derived from the 20 populations examined. Pairwise Euclidean distances were computed to generate a distance matrix, and hierarchical cluster analysis was conducted using the unweighted pair-group method with arithmetic mean (UPGMA) to produce a dendrogram depicting inter-population karyotypic differentiation.

To evaluate potential non-independence among these karyotypic variables, pairwise Pearson correlation coefficients were calculated for 2*n*, TDL, CV_CI_, M_CA_, and CV_CL_, revealing substantial redundancy among several variables. In particular, CV_CI_ was strongly correlated with M_CA_ (r = 0.968) and CV_CL_ (r = 0.902), both exceeding the commonly used |r| > 0.8 threshold for high correlation. Moreover, CV_CI_ and M_CA_ both largely describe variation associated with centromere position, whereas M_CA_ and CV_CL_ represent complementary dimensions of intrachromosomal and interchromosomal karyotype asymmetry, respectively. Therefore, CV_CI_ should be excluded from the sensitivity PCoA to minimize redundant weighting of correlated karyotypic information and improve interpretability. Because chromosome number (2*n*), together with the basic chromosome number (*x*), is intrinsically associated with cytotype designation, the sensitivity PCoA excluded *x* and 2*n* as well as CV_CI_, and was therefore conducted using TDL, M_CA_, and CV_CL_. Additional sensitivity analyses using TDL + MCA and TDL + CVCL were performed to evaluate whether the major ordination pattern was dependent on variable selection.

### 4.6. Cytogeographic Analysis

Geographic locations of all documented populations were compiled from both the current sampling records and original published sources (Table S4) [6,9,10,11,16,17,18,19,33,44,45,67,68,69,70]. Each record was assigned to one of three cytotypes—A (2*n* = 14), B (2*n* = 15), or C (2*n* = 16)—based on cytogenetic evidence. All geographic coordinates were standardized to decimal degrees using the WGS84 datum. For historical records lacking precise collection coordinates, locality-based proxy coordinates were estimated solely for cartographic representation; these approximated coordinates are explicitly flagged in Table S4. The resulting coordinate dataset was integrated into a GIS-based mapping workflow to generate spatial visualizations of the geographic ranges and spatial partitioning of cytotypes A, B, and C. Populations of *L. traubii* with 2*n* = 14—whose taxonomic status remains controversial—were incorporated into the analysis [6,8,9]. Populations lacking documented collection localities or verifiable provenance were excluded. Previously reported South Korean population of *L. aurea* with 2*n* = 16 [71] were subsequently reclassified as *L. chinensis* [72], and thus were not included in the analysis.

## 5. Conclusions

This study provides a systematic molecular cytogenetic characterization of the three predominant natural cytotypes—A (2*n* = 14 = 8m + 6t/st), B (2*n* = 15 = 7m + 8t/st), and C (2*n* = 16 = 6m + 10t/st)—within the *L. aurea* complex. Karyotype asymmetry increases progressively from cytotype A to B to C. PCoA and UPGMA dendrograms based on six karyotypic parameters consistently resolve substantial karyotypic differentiation between cytotype A and the other two cytotypes. Multivariate karyomorphometric analyses and comparative assessment of DAPI banding and rDNA FISH patterns reveal that cytotype A exhibits high inter-population karyotypic uniformity, cytotype C displays pronounced intra-cytotypic karyotypic polymorphism, and cytotype B shows more conspicuous chromosomal heteromorphism. Integrated cytogenetic evidence supports a working hypothesis that karyotype C represents the putative ancestral karyotype of the *L. aurea* complex, while karyotypes A and B originated from karyotype C through two and one Robertsonian fusion events, respectively—thereby implying two independent, parallel evolutionary pathways: C → A and C → B. The evolutionary biogeographic framework for this species complex—derived from hypothesis-generating, integrative inference—propose that cytotype A originated from a cytotype C population distributed along the Sichuan–Chongqing–Hubei corridor, whereas cytotype B arose from a geographically isolated cytotype C population restricted to the eastern Nanling refuge. Quaternary climate oscillations, topographic barriers, and ecological niche divergence acted synergistically to shape the current parapatric distribution pattern: cytotype C is predominantly distributed across low-latitude montane regions of southwestern and southern China; cytotype A predominates in subtropical inland mountain ranges of central China and offshore islands of East Asia; and cytotype B is narrowly restricted to the ecological transition zone of the Nanling Mountains. The hypotheses proposed in this study requires rigorous validation through an integrative approach combining molecular cytogenetics, molecular phylogenomics, population genomics and phylogeography, based on a comprehensive sample set with sufficient sampling density.

## Figures and Tables

**Table 1:** Karyotypic characteristics of 20 populations of the *Lycoris aurea* complex.

Population	Karyotype Formula	TDL (μm)	RRL	CI ± SE	CV_CI_	M_CA_	CV_CL_	Stebbins’ Type
SCXH	2*n* = 14 = 8m + 6t	236.23	4.36–9.96	30.20 ± 19.86	65.75	39.60	32.04	2B
SCDJY	2*n* = 14 = 8m + 6t	206.54	3.84–10.32	30.23 ± 20.93	69.25	39.55	35.78	2B
GDRY	2*n* = 14 = 8m + 1st + 5t	209.62	3.99–10.66	31.61 ± 19.18	60.68	36.78	32.50	2B
HNYL	2*n* = 14 = 8m + 1st + 5t	210.09	4.32–10.10	31.42 ± 18.19	57.88	37.16	33.43	2B
HNTD	2*n* = 14 = 8m + 6t	194.95	4.23–10.17	31.67 ± 19.65	62.06	36.67	32.45	2B
CQFD	2*n* = 14 = 8m + 1st + 5t	187.26	4.24–9.77	31.55 ± 19.10	60.55	36.91	31.23	2B
HBES	2*n* = 14 = 8m + 1st + 5t	220.85	4.05–10.01	31.03 ± 18.98	61.17	37.94	34.67	2B
ZJSZ	2*n* = 14 = 8m + 6t	184.74	4.12–9.79	30.59 ± 20.16	65.90	38.81	34.58	2B
FJHA	2*n* = 15 = 7m + 8t	224.91	3.73–10.39	26.58 ± 21.13	79.48	46.84	37.71	3B
GDGZ	2*n* = 15 = 7m + 2st + 6t	259.78	4.12–9.58	26.89 ± 20.69	76.95	46.21	35.78	3B
JXQN	2*n* = 15 = 7m + 8t	171.96	3.67–10.40	25.84 ± 20.98	81.21	48.33	38.32	3B
JSJR	2*n* = 16 = 6m + 1st + 9t	220.79	3.66–9.92	24.66 ± 18.81	76.25	50.67	41.44	3B
GXBM	2*n* = 16 = 6m + 10t	242.62	3.45–9.88	22.98 ± 19.69	85.68	54.03	41.38	3B
GXTL	2*n* = 16 = 6m + 10t	273.09	3.79–10.22	22.69 ± 20.15	88.80	54.61	39.81	3B
JSYX	2*n* = 16 = 6m + 10t	259.03	3.99–9.87	23.86 ± 19.30	80.87	52.28	40.86	3B
SCEMS	2*n* = 16 = 6m + 10t	200.08	3.99–10.18	23.19 ± 20.35	87.78	53.63	41.54	3B
AHHS	2*n* = 16 = 6m + 10t	199.97	4.06–10.26	24.10 ± 19.41	80.56	51.80	40.06	3B
CQCK	2*n* = 16 = 6m + 10t	204.74	3.57–10.39	22.26 ± 19.64	88.21	55.48	44.84	3B
SXMX	2*n* = 16 = 6m + 10t	213.18	3.69–9.83	22.48 ± 21.15	94.09	55.04	41.10	3B
HNTB	2*n* = 16 = 6m + 10t	164.64	3.79–9.73	22.99 ± 20.97	91.21	54.01	41.82	3B

Notes: TDL, the total diploid chromosome length; RRL, range of chromosome relative length; CI, mean centromeric index; CV_CI_, coefficient of variation of centromeric index; M_CA_, mean centromeric asymmetry; CV_CL_, coefficient of variation of chromosome lengths; Stebbins’ types, karyotype asymmetry category of Stebbins.

**Table 2:** The number and position of fluorescent bands and 45S rDNA sites in 20 populations of the *Lycoris aurea* complex.

Population	DAPI Band			CPD Band/45S rDNA Site		
	Number	Position ^#^	Amount (%) *	Number ^†^	Position ^#^	Amount (%) *
SCXH	9	CENs of pair 2 and one member of pair 3; (CENs + L-PCENs) of pairs 5–7	3.11	8/10	L-PCENs of pair 4; S of pairs 5–7; L-PROXs of pair 5 ^※^	2.43
SCDJY	8	CENs of pair 2; (CENs + L-PCENs) of pairs 5–7	2.24	/10	S-PCENs of pair 4; S of pairs 5–7; L-PROXs of pair 7 ^※^	1.78 ^§^
GDRY	9	CENs of pair1 and one member of pair 2; (CENs + L-PCENs) of pairs 5–7	4.66	10/10	L/S-PCENs of pair 2; S of pairs 5–7; L-PROXs of pair 6	3.02
HNYL	6	(CENs + L-PCENs) of pairs 5–7	2.21	8/10	L-PCENs of pair 4; S of pairs 5–7; L-PROXs of pair 7 ^※^	2.66
HNTD	6	(CENs + L-PCENs) of pairs 5–7	2.07	8/10	L-PCENs of pair 4; S of pairs 5–7; L-PROXs of pair 7 ^※^	2.21
CQFD	6	(CENs + L-PCENs) of pairs 5–7	2.21	10/10	L-PCENs of pair 4; S of pairs 5–7; L-PROXs of pair 7	2.56
HBES	5	(CENs + L-PCENs) of pairs 5, 6 and one member of pair 7	1.53	8/10	L-PCENs of pair 4; S of pairs 5–7; L-PROXs of pair 6 ^※^	2.15
ZJSZ	6	(CENs + L-PCENs) of pair 5–7	2.16	9/9	L-PCEN of pair 4; S of pairs 5, 6 and one member of pair 7; L-PROXs of pair 7	1.49
FJHA	4	CEN and S-PCEN of chr. 6; (CENs + L-PCENs) of chr. 9, 13	0.82	/9	S-PCEN of chr. 5, 6; S of chr. 8–11, 13–15	2.06 ^§^
GDGZ	3	CEN of chr. 7; (CENs + L-PCENs) of chr. 8, 9	0.64	8/9	S-PCENs of chr. 4 ^※^, 7; S of chr. 8–14	2.68
JXQN	2	L-PCEN of chr. 5; (CEN + L-PCEN) of chr. 9	0.56	5/6	L-PCENs of chr. 4 ^※^; S of chr. 8, 10–13	1.78
JSJR	5	L-PCEN of pair 1; (CENs + L-PCENs) of pair 4 and one member of pair 6	1.40	/14	CENs of pair 2, 3; S of pairs 4–8	4.59 ^§^
GXBM	12	CENs of one member of pairs 2 and one member of pair 3; (CENs + L-PCENs) of pairs 4–8	3.18	10/11	S of pairs 4–8; L-PROX of one member of pair 7 ^※^	3.00
GXTL	14	CENs of pairs 1, 3; (CENs + L-PCENs) of pairs 4–8	2.99	10/	S of pairs 4–8	2.10
JSYX	5	S/L-PCEN of pair 3; (CENs + L-PCENs) of pair 6 and one member of pair 4	1.17	10/	S of pairs 4–8	3.13
SCEMS	12	S-PCENs of pairs 1, 3; (CENs + L-PCENs) of pairs 4–6, 8	2.20	10/10	S of pairs 4–8	2.50
AHHS	6	S-PCEN of pair 1; (CENs + L-PCENs) of pairs 5, 6	1.48	10/10	S of pairs 4–8	3.23
CQCK	4	L-PCEN of one member of pair 2, S-PCEN of one member of pair 3; (CENs + L-PCENs) of pair 4	0.84	10/11	L-PCEN of one member of pair 1 ^※^; S of pairs 4–8	1.98
SXMX	2	(CENs + L-PCENs) of pair 4	0.38	10/11	L-PCEN of one member of pair 2 ^※^; S of pairs 4–8	2.31
HNTB	2	(CENs + L-PCENs) of pair 4	0.33	9/11	S-PCEN of one member of pair 1 ^※^; S of pairs 5–8, one member of pair 4, and another member of pair 4 ^※^	2.36

**^#^** S and L represent the short and long arms, respectively; CEN, PCEN, PROX represent the centromeric, pericentromeric, and proximal positions, respectively; for populations with 2*n* = 14 and 2*n* = 16 chromosomes are arranged by homologous pairs (designated as pair); for the 2*n* = 15 populations, chromosomes were sorted individually (designated as chr.). * Amount of DAPI bands and CPD bands (45S rDNA) in the genome, expressed as a percentage of the total diploid chromosome length (TDL). ^†^ The numbers to the left and right of the slash represent the counts of CPD bands and 45S rDNA sites, respectively. ^※^ 45S rDNA sites that failed to resolve as distinct CPD bands following CPD staining. ^§^ The assay quantifies the 45S rDNA hybridization signal, not the CPD band.

## Data Availability

Data are contained within the article and Supplementary Materials. Further inquiries can be directed to the corresponding author.

## Supplementary Materials

The following supporting information can be downloaded at: https://www.mdpi.com/article/10.3390/plants15192993/s1, Table S1: The plant materials; Table S2: Chromosome measurements of 20 populations of the *Lycoris aurea* complex; Table S3: The full nucleotide sequences of the oligonucleotide probes; Table S4: Geographical distribution of documented populations of the *Lycoris aurea* complex; Figure S1: Principal coordinate analysis (PCoA) of 20 populations of the *Lycoris aurea* complex based on three karyotypic parameters: TDL, M_CA_ and CV_CL_.

## Abbreviations

The following abbreviations are used in this manuscript:

FISH	Fluorescence In Situ Hybridization
CPD	Combined PI (propidium iodide) and DAPI (4,6-diamidino-2-phenylindole)
TDL	the Total Diploid Chromosome Length
TCL	the Total Length of Haploid Complement
CI	Mean Centromeric Index
RRL	Range of Chromosome Relative Length
M_CA_	Mean Centromeric Asymmetry
CV_CL_	Coefficient of Variation of Chromosome Length
CV_CI_	Coefficient of Variation of Centromeric Index
LIG	Last Interglacial
LGM	Last Glacial Maximum
