# Supplementary material for: Intraspecific Karyotypic Differentiation in the Lycoris aurea Complex Revealed by Fluorochrome Banding and rDNA Fluorescence In Situ Hybridization
Source: Plants (Basel). 2026 Sep 30;15(19):2993. doi: 10.3390/plants15192993 (PMC13667273; doi:10.3390/plants15192993)
Supplement: Supplementary file 1 [file plants-15-02993-s001.zip › plants-4546736-supplementary.pdf]

Supplementary Files

Table S1. The plant materials.

Population	Collection location	Longitude and latitude
SCXH	Xuanhan County, Sichuan Province	108°15′E, 31°40′N
SCDJY	Dujiangyan City, Sichuan Province	103°49′E, 30°93N
GDRY	Ruyuan County, Guangdong Province	113°29′E, 25°01′N
HNYL	Yuanling County, Hunan Province	110°37′E, 28°44′N
HNTD	Tongdao County, Hunan Province	109°47′E, 26°09′N
CQFD	Fengdu County, Chongqing Municipality	107°42′E, 30°11′N
HBES	Enshi City, Hubei Province	109°18′E, 30°42′N
ZJSZ	Shengzhou County, Zhejiang Province	120°57′E, 29°38′N
FJHA	Hui 'an County, Fujian Province	119°00′E, 24°94′N
GDGZ	Guangzhou City, Guangdong Province	113°37′E, 23°18′N
JXQN	Quannan County, Jiangxi Province	114°35′E, 24°47′N
JSJR	Jurong County, Jiangsu Province	119°05′E,32°08′N
GXBM	Bama County, Guangxi Zhuang Autonomous Region	107°05′E, 24°14′N
GXTL	Tianlin County, Guangxi Zhuang Autonomous Region	105°42′E, 24°35′N
SCEMS	Emeishan City, Sichuan Province	103°44′E, 29°56′N
AHHS	Huangshan City, Anhui Province	118°10'E, 30°12'N
JSYX	Yixing City, Jiangsu Province	119°69′E, 31°34′N
CQCK	Chengkou County, Chongqing Municipality	108°19′E, 31°74′N
SXMX	Meixian County, Shaanxi Province	107°42′E, 34°05′N
HNTB	Tongbai County, Henan Province	113°34′E, 32°35′N

Table S2. Chromosome measurements of 20 populations of the *Lycoris aurea* complex.

Population	Chr. No.	Relative length (%)†			Arm ratio	Chr. type	DAPI band size†	CPD band size†
		Short arm	Long arm	Total				
SCXH	1	4.73	5.23	9.96	1.11	m	0.00	0.00
	1'	4.54	5.23	9.77	1.15	m	0.00	0.00
	2	4.36	4.73	9.09	1.09	m	0.25	0.00
	2'	4.11	5.10	9.21	1.24	m	0.25	0.00
	3	4.42	4.73	9.15	1.07	m	0.25	0.00
	3'	4.36	4.42	8.77	1.01	m	0.25	0.00
	4	3.67	4.36	8.03	1.19	m	0.00	0.25
	4'	3.48	4.54	8.03	1.30	m	0.00	0.25
	5	0.50	4.48	4.98	9.00	t	0.25	0.44
	5'	0.44	4.17	4.60	9.57	t	0.25	0.37
	6	0.31	4.48	4.79	14.40	t	0.56	0.25
	6'	0.31	4.29	4.60	13.80	t	0.44	0.25
	7	0.44	4.23	4.67	9.71	t	0.25	0.37
	7'	0.31	4.04	4.36	13.00	t	0.37	0.25
	Total	35.97	64.03	100.00			3.11	2.43
SCDJY	1	4.70	5.05	9.75	1.08	m	0.00	0.00
	1'	4.77	5.55	10.32	1.16	m	0.00	0.00
	2	4.91	5.05	9.96	1.03	m	0.14	0.00
	2'	4.48	5.62	10.11	1.25	m	0.14	0.00
	3	4.20	4.27	8.47	1.02	m	0.00	0.00
	3'	4.34	4.63	8.97	1.07	m	0.00	0.00
	4	3.70	3.84	7.54	1.04	m	0.00	0.21
	4'	3.77	4.41	8.19	1.17	m	0.00	0.14
	5	0.36	4.63	4.98	13.00	t	0.43	0.28
	5'	0.28	4.70	4.98	16.50	t	0.28	0.21
	6	0.36	3.84	4.20	10.80	t	0.18	0.28
	6'	0.28	4.48	4.77	15.75	t	0.57	0.21
	7	0.28	3.63	3.91	12.75	t	0.21	0.21
	7'	0.28	3.56	3.84	12.50	t	0.28	0.21
	Total	36.73	63.27	100.00			2.24	1.78
GDRY	1	4.91	5.12	10.03	1.04	m	0.28	0.00
	1'	5.05	5.61	10.66	1.11	m	0.28	0.00
	2	3.86	5.47	9.33	1.42	m	0.00	0.28
	2'	4.28	4.35	8.63	1.02	m	0.98	0.14
	3	4.49	4.63	9.12	1.03	m	0.00	0.00
	3'	4.07	4.14	8.20	1.02	m	0.00	0.00
	4	3.51	3.86	7.36	1.10	m	0.00	0.00
	4'	3.79	4.42	8.20	1.17	m	0.00	0.00
	5	0.77	4.91	5.68	6.36	st	0.63	0.70
	5'	0.49	4.56	5.05	9.29	t	0.67	0.42
	6	0.49	4.00	4.49	8.14	t	0.46	0.42
	6'	0.56	4.28	4.84	7.63	t	0.53	0.49

	7	0.28	4.14	4.42	14.75	t	0.42	0.21
	7'	0.42	3.58	4.00	8.50	t	0.42	0.35
	Total	36.96	63.04	100.00			4.66	3.02
HNYL	1	4.18	5.73	9.92	1.37	m	0.00	0.00
	1'	4.45	5.65	10.10	1.27	m	0.00	0.00
	2	4.49	4.74	9.23	1.06	m	0.00	0.00
	2'	4.34	4.78	9.12	1.10	m	0.00	0.00
	3	4.13	4.56	8.69	1.10	m	0.00	0.00
	3'	4.33	4.85	9.18	1.12	m	0.00	0.00
	4	3.80	4.30	8.10	1.13	m	0.00	0.13
	4'	4.01	4.34	8.36	1.08	m	0.00	0.13
	5	0.58	4.22	4.81	7.25	t	0.40	0.46
	5'	0.41	4.17	4.58	10.16	t	0.24	0.33
	6	0.49	4.03	4.52	8.22	t	0.52	0.37
	6'	0.61	4.09	4.70	6.72	st	0.41	0.52
	7	0.52	3.81	4.33	7.38	t	0.34	0.38
	7'	0.48	3.89	4.37	8.17	t	0.30	0.33
	Total	36.82	63.18	100.00			2.21	2.66
HNTD	1	4.73	4.77	9.50	1.01	m	0.00	0.00
	1'	5.04	5.13	10.17	1.02	m	0.00	0.00
	2	4.68	4.73	9.41	1.01	m	0.00	0.00
	2'	4.46	5.00	9.45	1.12	m	0.00	0.00
	3	4.05	4.59	8.64	1.13	m	0.00	0.00
	3'	4.28	4.32	8.60	1.01	m	0.00	0.00
	4	3.92	4.50	8.42	1.15	m	0.00	0.09
	4'	3.56	4.41	7.97	1.24	m	0.00	0.09
	5	0.41	4.59	5.00	11.33	t	0.54	0.27
	5'	0.45	4.59	5.04	10.20	t	0.32	0.36
	6	0.54	4.10	4.64	7.58	t	0.36	0.36
	6'	0.45	4.10	4.55	9.10	t	0.36	0.32
	7	0.54	3.87	4.41	7.17	t	0.23	0.45
	7'	0.36	3.87	4.23	10.75	t	0.27	0.27
	Total	37.44	62.56	100.00			2.07	2.21
CQFD	1	4.77	4.99	9.77	1.05	m	0.00	0.00
	1'	4.68	4.95	9.63	1.06	m	0.00	0.00
	2	4.51	4.73	9.24	1.05	m	0.00	0.00
	2'	4.42	4.51	8.93	1.02	m	0.00	0.00
	3	4.37	4.64	9.01	1.06	m	0.00	0.00
	3'	4.07	4.90	8.97	1.21	m	0.00	0.00
	4	3.40	4.73	8.13	1.39	m	0.00	0.18
	4'	3.76	4.20	7.95	1.12	m	0.00	0.18
	5	0.44	4.46	4.90	10.10	t	0.35	0.31
	5'	0.80	4.46	5.26	5.61	st	0.57	0.62
	6	0.40	4.29	4.68	10.78	t	0.35	0.31
	6'	0.40	4.20	4.60	10.56	t	0.31	0.31
	7	0.44	3.80	4.24	8.60	t	0.27	0.31

	7'	0.49	4.20	4.68	8.64	t	0.35	0.35
	Total	36.94	63.06	100.00			2.21	2.56
HBES	1	4.94	5.08	10.02	1.03	m	0.00	0.00
	1'	4.54	5.23	9.77	1.15	m	0.00	0.00
	2	4.39	4.99	9.38	1.13	m	0.00	0.00
	2'	4.39	4.99	9.38	1.13	m	0.00	0.00
	3	4.24	4.74	8.98	1.12	m	0.00	0.00
	3'	4.15	4.74	8.88	1.14	m	0.00	0.00
	4	3.95	4.54	8.49	1.15	m	0.00	0.10
	4'	3.80	4.69	8.49	1.23	m	0.00	0.10
	5	0.49	4.15	4.64	8.40	t	0.44	0.35
	5'	0.49	4.34	4.84	8.80	t	0.35	0.35
	6	0.59	3.95	4.54	6.67	st	0.25	0.49
	6'	0.35	3.80	4.15	11.00	t	0.25	0.20
	7	0.35	4.05	4.39	11.71	t	0.25	0.25
	7'	0.39	3.65	4.05	9.25	t	0.00	0.32
	Total	37.07	62.93	100.00			1.53	2.15
ZJSZ	1	4.43	5.36	9.78	1.21	m	0.00	0.00
	1'	4.74	4.94	9.68	1.04	m	0.00	0.00
	2	4.79	4.94	9.73	1.03	m	0.00	0.00
	2'	4.69	4.84	9.53	1.03	m	0.00	0.00
	3	4.22	5.10	9.32	1.21	m	0.00	0.00
	3'	4.38	4.48	8.86	1.02	m	0.00	0.00
	4	3.66	4.38	8.03	1.20	m	0.00	0.10
	4'	3.76	4.48	8.24	1.19	m	0.00	0.10
	5	0.36	4.22	4.58	11.71	t	0.46	0.15
	5'	0.36	4.43	4.79	12.29	t	0.36	0.15
	6	0.36	3.91	4.27	10.86	t	0.31	0.21
	6'	0.51	4.07	4.58	7.90	t	0.36	0.41
	7	0.41	3.81	4.22	9.25	t	0.26	0.36
	7'	0.15	4.22	4.38	27.33	t	0.41	0.00
	Total	36.82	63.18	100.00			2.16	1.49
FJHA	1	4.71	5.69	10.39	1.21	m	0.00	0.00
	2	4.84	5.10	9.93	1.05	m	0.00	0.00
	3	4.58	4.77	9.35	1.04	m	0.00	0.00
	4	4.44	4.51	8.95	1.01	m	0.00	0.00
	5	4.31	4.64	8.95	1.08	m	0.00	0.00
	6	4.05	4.31	8.37	1.06	m	0.33	0.00
	7	3.99	4.12	8.10	1.03	m	0.00	0.00
	8	0.52	5.36	5.88	10.25	t	0.00	0.46
	9	0.39	4.58	4.97	11.67	t	0.36	0.26
	10	0.33	4.38	4.71	13.40	t	0.00	0.26
	11	0.46	4.18	4.64	9.14	t	0.00	0.39
	12	0.16	3.99	4.15	24.40	t	0.00	0.00
	13	0.33	3.66	3.99	11.20	t	0.13	0.26
	14	0.23	3.66	3.89	16.00	t	0.00	0.16

	15	0.33	3.40	3.73	10.40	t	0.00	0.26
	Total	33.66	66.34	100.00			0.82	2.06
GDGZ	1	4.61	4.98	9.59	1.08	m	0.00	0.00
	2	4.28	5.20	9.48	1.21	m	0.00	0.00
	3	4.66	4.77	9.43	1.02	m	0.00	0.00
	4	4.61	4.77	9.37	1.03	m	0.00	0.00
	5	4.39	4.45	8.84	1.01	m	0.00	0.00
	6	4.23	4.28	8.52	1.01	m	0.00	0.00
	7	3.75	4.71	8.46	1.26	m	0.11	0.11
	8	0.37	4.82	5.20	12.86	t	0.32	0.27
	9	0.16	4.45	4.61	27.67	t	0.21	0.11
	10	0.59	4.02	4.61	6.82	st	0.00	0.54
	11	0.54	4.02	4.55	7.50	t	0.00	0.48
	12	0.64	3.91	4.55	6.08	st	0.00	0.59
	13	0.54	3.86	4.39	7.20	t	0.00	0.48
	14	0.16	4.12	4.28	25.67	t	0.00	0.11
	15	0.11	4.02	4.12	37.50	t	0.00	0.00
	Total	33.64	66.36	100.00			0.64	2.68
JXQN	1	5.05	5.35	10.40	1.06	m	0.00	0.00
	2	4.64	5.05	9.68	1.09	m	0.00	0.00
	3	4.59	4.79	9.38	1.04	m	0.00	0.00
	4	4.23	4.94	9.17	1.17	m	0.00	0.00
	5	3.98	4.99	8.97	1.26	m	0.25	0.00
	6	4.13	4.74	8.87	1.15	m	0.00	0.00
	7	4.03	4.18	8.21	1.04	m	0.00	0.00
	8	0.56	4.43	4.99	7.91	t	0.00	0.51
	9	0.15	4.69	4.84	30.67	t	0.31	0.00
	10	0.56	4.13	4.69	7.36	t	0.00	0.51
	11	0.36	4.28	4.64	12.00	t	0.00	0.31
	12	0.36	4.03	4.38	11.29	t	0.00	0.31
	13	0.20	4.13	4.33	20.25	t	0.00	0.15
	14	0.15	3.62	3.77	23.67	t	0.00	0.00
	15	0.15	3.52	3.67	23.00	t	0.00	0.00
	Total	33.13	66.87	100.00			0.56	1.78
JSJR	1	4.73	5.19	9.92	1.10	m	0.13	0.00
	1'	4.79	4.86	9.65	1.01	m	0.13	0.00
	2	4.39	5.26	9.65	1.20	m	0.00	0.13
	2'	4.26	4.66	8.92	1.09	m	0.00	0.13
	3	4.06	4.46	8.52	1.10	m	0.00	0.20
	3'	4.99	4.99	9.99	1.00	m	0.00	0.20
	4	0.33	4.26	4.59	12.80	t	0.33	0.27
	4'	0.33	4.39	4.73	13.20	t	0.40	0.27
	5	0.67	3.93	4.59	5.90	st	0.00	0.60
	5'	0.53	3.93	4.46	7.38	t	0.00	0.47
	6	0.53	4.06	4.59	7.63	t	0.00	0.47
	6'	0.40	3.99	4.39	10.00	t	0.40	0.33

	7	0.47	3.60	4.06	7.71	t	0.00	0.40
	7'	0.47	3.60	4.06	7.71	t	0.00	0.40
	8	0.40	3.26	3.66	8.17	t	0.00	0.33
	8'	0.47	3.73	4.19	8.00	t	0.00	0.40
	Total	31.82	68.18	100.00			1.40	4.59
GXBM	1	4.67	5.21	9.88	1.12	m	0.00	0.00
	1'	4.67	5.09	9.75	1.09	m	0.00	0.00
	2	4.54	5.15	9.69	1.13	m	0.36	0.00
	2'	4.54	4.79	9.33	1.05	m	0.00	0.00
	3	4.12	4.79	8.91	1.16	m	0.00	0.00
	3'	4.36	4.67	9.03	1.07	m	0.18	0.00
	4	0.48	4.54	5.03	9.38	t	0.48	0.42
	4'	0.36	4.54	4.91	12.50	t	0.42	0.30
	5	0.42	4.24	4.67	10.00	t	0.27	0.36
	5'	0.36	4.48	4.85	12.33	t	0.36	0.30
	6	0.42	4.06	4.48	9.57	t	0.27	0.36
	6'	0.30	3.76	4.06	12.40	t	0.12	0.24
	7	0.42	3.64	4.06	8.57	t	0.21	0.36
	7'	0.36	3.64	4.00	10.00	t	0.18	0.30
	8	0.21	3.45	3.67	16.29	t	0.15	0.15
	8'	0.24	3.45	3.70	14.25	t	0.15	0.18
	Total	30.51	69.49	100.00			3.18	3.00
GXTL	1	5.05	5.17	10.22	1.02	m	0.11	0.00
	1'	4.76	4.82	9.59	1.01	m	0.11	0.00
	2	4.65	4.82	9.47	1.04	m	0.00	0.00
	2'	4.25	5.34	9.59	1.26	m	0.00	0.00
	3	4.25	4.48	8.73	1.05	m	0.11	0.00
	3'	3.67	4.42	8.09	1.20	m	0.17	0.00
	4	0.46	4.76	5.22	10.38	t	0.34	0.29
	4'	0.29	4.48	4.76	15.60	t	0.40	0.17
	5	0.29	4.76	5.05	16.60	t	0.23	0.17
	5'	0.29	4.19	4.48	14.60	t	0.23	0.20
	6	0.40	4.02	4.42	10.00	t	0.23	0.23
	6'	0.40	3.85	4.25	9.57	t	0.23	0.23
	7	0.34	3.67	4.02	10.67	t	0.23	0.23
	7'	0.40	3.79	4.19	9.43	t	0.29	0.23
	8	0.34	3.79	4.13	11.00	t	0.17	0.23
	8'	0.17	3.62	3.79	21.00	t	0.11	0.11
	Total	30.02	69.98	100.00			2.99	2.10
JSYX	1	4.68	5.09	9.76	1.09	m	0.00	0.00
	1'	4.88	4.98	9.87	1.02	m	0.00	0.00
	2	4.63	4.78	9.41	1.03	m	0.00	0.00
	2'	4.68	4.78	9.46	1.02	m	0.00	0.00
	3	3.81	4.78	8.59	1.25	m	0.15	0.00
	3'	4.42	4.98	9.41	1.13	m	0.15	0.00
	4	0.46	4.27	4.73	9.33	t	0.25	0.28

	4'	0.41	4.22	4.63	10.38	t	0.00	0.36
	5	0.41	3.92	4.32	9.63	t	0.00	0.31
	5'	0.46	4.17	4.63	9.11	t	0.00	0.41
	6	0.36	4.12	4.47	11.57	t	0.31	0.15
	6'	0.41	3.92	4.32	9.63	t	0.31	0.23
	7	0.31	3.97	4.27	13.00	t	0.00	0.25
	7'	0.46	3.61	4.07	7.89	t	0.00	0.41
	8	0.38	3.61	3.99	9.47	t	0.00	0.33
	8'	0.46	3.61	4.07	7.89	t	0.00	0.41
	Total	31.20	68.80	100.00			1.17	3.13
SCEMS	1	4.99	5.19	10.18	1.04	m	0.15	0.00
	1'	4.60	5.09	9.69	1.11	m	0.10	0.00
	2	4.41	4.55	8.96	1.03	m	0.00	0.00
	2'	4.75	5.29	10.04	1.11	m	0.00	0.00
	3	4.60	4.70	9.30	1.02	m	0.20	0.00
	3'	4.16	4.36	8.52	1.05	m	0.15	0.00
	4	0.34	4.41	4.75	12.86	t	0.24	0.29
	4'	0.44	4.41	4.85	10.00	t	0.24	0.34
	5	0.29	4.11	4.41	14.00	t	0.10	0.20
	5'	0.34	4.01	4.36	11.71	t	0.15	0.20
	6	0.44	3.92	4.36	8.89	t	0.49	0.29
	6'	0.29	4.06	4.36	13.83	t	0.20	0.15
	7	0.44	3.53	3.97	8.00	t	0.00	0.34
	7'	0.39	3.82	4.21	9.75	t	0.00	0.34
	8	0.24	3.82	4.06	15.60	t	0.10	0.20
	8'	0.22	3.77	3.99	17.11	t	0.10	0.15
	Total	30.97	69.03	100.00			2.20	2.50
AHHS	1	4.74	5.52	10.26	1.16	m	0.18	0.00
	1'	4.79	5.11	9.90	1.07	m	0.16	0.00
	2	4.64	4.85	9.48	1.04	m	0.00	0.00
	2'	4.38	4.58	8.96	1.05	m	0.00	0.00
	3	4.17	4.38	8.54	1.05	m	0.00	0.00
	3'	4.32	4.53	8.86	1.05	m	0.00	0.00
	4	0.42	4.27	4.69	10.25	t	0.00	0.36
	4'	0.42	4.22	4.64	10.13	t	0.00	0.36
	5	0.36	4.27	4.64	11.71	t	0.31	0.21
	5'	0.39	4.17	4.56	10.67	t	0.31	0.21
	6	0.52	4.01	4.53	7.70	t	0.26	0.31
	6'	0.42	4.01	4.43	9.63	t	0.26	0.31
	7	0.42	3.65	4.06	8.75	t	0.00	0.36
	7'	0.36	3.86	4.22	10.57	t	0.00	0.31
	8	0.42	3.70	4.12	8.88	t	0.00	0.36
	8'	0.47	3.65	4.12	7.78	t	0.00	0.42
	Total	31.23	68.77	100.00			1.48	3.23
CQCK	1	4.58	5.81	10.39	1.27	m	0.00	0.00
	1'	4.58	5.31	9.89	1.16	m	0.00	0.00

	2	4.86	5.20	10.06	1.07	m	0.22	0.00
	2'	4.30	5.14	9.44	1.19	m	0.00	0.00
	3	4.53	4.92	9.44	1.09	m	0.11	0.00
	3'	4.30	4.64	8.94	1.08	m	0.00	0.00
	4	0.36	4.81	5.17	13.23	t	0.28	0.17
	4'	0.34	4.19	4.53	12.50	t	0.22	0.17
	5	0.39	4.41	4.81	11.29	t	0.00	0.34
	5'	0.39	3.91	4.30	10.00	t	0.00	0.28
	6	0.22	3.80	4.02	17.00	t	0.00	0.17
	6'	0.28	3.91	4.19	14.00	t	0.00	0.17
	7	0.22	3.69	3.91	16.50	t	0.00	0.17
	7'	0.28	3.41	3.69	12.20	t	0.00	0.17
	8	0.39	3.19	3.58	8.14	t	0.00	0.25
	8'	0.28	3.35	3.63	12.00	t	0.00	0.11
	Total	30.32	69.68	100.00			0.84	1.98
SXMX	1	4.72	4.89	9.61	1.03	m	0.00	0.00
	1'	4.89	4.94	9.83	1.01	m	0.00	0.00
	2	4.56	4.99	9.55	1.10	m	0.00	0.00
	2'	4.72	4.99	9.72	1.06	m	0.00	0.00
	3	4.67	4.99	9.66	1.07	m	0.00	0.00
	3'	3.96	4.02	7.98	1.01	m	0.00	0.00
	4	0.43	4.67	5.10	10.75	t	0.22	0.27
	4'	0.27	4.67	4.94	17.20	t	0.16	0.22
	5	0.43	4.23	4.67	9.75	t	0.00	0.38
	5'	0.33	4.13	4.45	12.67	t	0.00	0.27
	6	0.27	4.07	4.34	15.00	t	0.00	0.22
	6'	0.22	4.13	4.34	19.00	t	0.00	0.16
	7	0.22	3.80	4.02	17.50	t	0.00	0.16
	7'	0.27	3.85	4.13	14.20	t	0.00	0.22
	8	0.33	3.64	3.96	11.17	t	0.00	0.27
	8'	0.16	3.53	3.69	21.67	t	0.00	0.14
	Total	30.46	69.54	100.00			0.38	2.31
HNTB	1	4.67	5.05	9.73	1.08	m	0.00	0.00
	1'	4.73	5.00	9.73	1.06	m	0.00	0.00
	2	4.51	4.78	9.29	1.06	m	0.00	0.00
	2'	4.67	4.78	9.45	1.02	m	0.00	0.00
	3	4.84	4.84	9.67	1.00	m	0.00	0.00
	3'	4.51	4.56	9.07	1.01	m	0.00	0.00
	4	0.55	4.45	5.00	8.10	t	0.16	0.44
	4'	0.22	4.73	4.95	21.50	t	0.16	0.05
	5	0.38	4.01	4.40	10.43	t	0.00	0.27
	5'	0.33	4.12	4.45	12.50	t	0.00	0.22
	6	0.38	3.85	4.23	10.00	t	0.00	0.33
	6'	0.38	3.63	4.01	9.43	t	0.00	0.33
	7	0.11	3.68	3.79	33.50	t	0.00	0.11
	7'	0.11	4.18	4.29	38.00	t	0.00	0.11

8	0.38	3.74	4.12	9.71	t	0.00	0.33
8'	0.33	3.52	3.85	10.67	t	0.00	0.16
Total	31.10	68.90	100.00			0.33	2.36

Notes: m, metacentric; st, subtelocentric; t, telocentric; †, percentage of the total diploid chromosome length (TDL); Chr., Chromosome.

Table S3. The full nucleotide sequences of the oligonucleotide probes.

Primer	Sequence and fluorochrome label	Base number
oligo 5S-1	6-FAM-5′GGATGCGAT CATACCAGCA CTAATGCACC GGATCCCATC AGAACTCCGC AGTTAAGCGT3′	59
oligo 5S-2	6-FAM-5′GCTTGGGCGA GAGTAGTACTAGGATGGGTG ACCTCTCGGG AAATCCTCGT GTTGCATC3′	58
oligo 45S-1	TAMRA-5′AAAACGACTC TCGGCAACGG ATATCTCGGC TCTCGCATCG ATGAAGAACG TAGCGAAAT3′-TAMRA	59
oligo 45S-2	TAMRA-5′TACCTGGTTG ATCCTGCCAG TAGTCATATG CTTGTCTCAA AGATTAAGCC ATGCATGTG3′-TAMRA	59
oligo 45S-3	TAMRA-5′CCCGCTGAGT TTAAGCATAT CAATAAGCGG AGGAAAAGAA ACTAACAAGG ATTCCCTTA3′-TAMRA′	59

Note: 6-FAM and TAMRA refer to 6-carboxyl fluorescein and 6-carboxyl-tetramethyl rhodamine, respectively.

Table S4. Geographical distribution of documented populations of the *Lycoris aurea* complex.

No	Cytotype	Locality	Longitude	Latitude	Source	Author
1	A	Fengdu, Chongqing	107.70	30.18	This study	This study
2	A	Jinfo Mountain, Chongqing	107.11	29.05	Published record	Shu et al. 2010 [69]
3	A	Pengshui, Chongqing	108.20	29.50	Published record	Wang et al. 2022 [6]
4	A	Ruyuan, Guangdong	113.48	25.02	This study	This study
5	A	Baise, Guangxi	106.24	24.28	Published record	Wang et al. 2022 [6]
6	A	Baise, Guangxi	105.63	24.50	Published record	Wang et al. 2022 [6]
7	A	Guilin, Guangxi	110.07	25.37	Published record	Wang et al. 2022 [6]
8	A	Guilin, Guangxi	110.32	25.68	Published record	Wang et al. 2022 [6]
9	A	Liuzhou, Guangxi	109.40	25.22	Published record	Wang et al. 2022 [6]
10	A	Dushan, Guizhou	107.57	25.87	Published record	Jiang et al. 2024 [11]
11	A	Qiandongnan, Guizhou	107.80	27.13	Published record	Wang et al. 2022 [6]
12	A	Tongren, Guizhou	108.75	27.77	Published record	Wang et al. 2022 [6]
13	A	Badong, Hubei	110.32	31.17	Published record	Jiang et al. 2024 [11]
14	A	Enshi, Hubei	109.61	29.68	Published record	Wang et al. 2022 [6]
15	A	Enshi, Hubei	109.30	30.70	This study	This study
16	A	Fangxian, Hubei	110.85	32.84	Published record	Jiang et al. 2024 [11]
17	A	Shiyan, Hubei	111.04	32.48	Published record	Wang et al. 2022 [6]
18	A	Wuhan, Hubei	114.31	30.52	Published record	Ren et al. 2026 [70]
19	A	Yichang, Hubei	110.67	30.20	Published record	Wang et al. 2022 [6]
20	A	Yichang, Hubei	110.88	31.23	Published record	Wang et al. 2022 [6]
21	A	Yichang, Hubei	111.75	31.17	Published record	Wang et al. 2022 [6]
22	A	Changde, Hunan	111.25	29.53	Published record	Wang et al. 2022 [6]
23	A	Huaihua, Hunan	109.22	27.27	Published record	Wang et al. 2022 [6]
24	A	Huaihua, Hunan	109.17	27.37	Published record	Wang et al. 2022 [6]
25	A	Shaoyang, Hunan	110.24	26.33	Published record	Wang et al. 2022 [6]
26	A	Tongdao, Hunan	109.78	26.15	This study	This study
27	A	Xiangxi, Hunan	109.38	28.58	Published record	Wang et al. 2022 [6]

No	Cytotype	Locality	Longitude	Latitude	Source	Author
28	A	Xinning, Hunan	110.35	26.75	Published record	Jiang et al. 2024 [11]
29	A	Yongzhou, Hunan	111.56	25.50	Published record	Wang et al. 2022 [6]
30	A	Yuanling, Hunan	110.45	28.85	Published record	Wang et al. 2022 [6]
31	A	Yuanling, Hunan	110.62	28.73	Published record	Quan et al. 2024 [18]
32	A	Yuanling, Hunan	110.62	28.73	This study	This study
33	A	Zhangjiajie, Hunan	110.52	29.00	Published record	Wang et al. 2022 [6]
34	A	Zhangjiajie, Hunan	110.49	29.35	Published record	Wang et al. 2022 [6]
35	A	Zhangjiajie, Hunan	110.42	29.38	Published record	Wang et al. 2022 [6]
36	A	Zhuzhou, Hunan	113.77	26.20	Published record	Wang et al. 2022 [6]
37	A	Osaka, Japan*	135.50	34.70	Published record	Ma and Tarumoto 2002 [45]
38	A	Ryukyu Islands	127.70	26.13	Published record	Kurita et al. 1987 [9]
39	A	Ryukyu Islands	124.19	24.40	Published record	Kurita et al. 1987 [9]
40	A	Ryukyu Islands	127.15	26.36	Published record	Kurita et al. 1987 [9]
41	A	Kagoshima, Japan	130.46	31.23	Published record	Wang et al. 2022 [6]
42	A	Chengdu, Sichuan*	104.07	30.67	Published record	Liu and Hsu 1989 [17]
43	A	Dujiangyan, Sichuan	103.56	30.87	Published record	Wang et al. 2022 [6]
44	A	Dujiangyan, Sichuan	103.49	30.93	This study	This study
45	A	Guangyuan, Sichuan	106.25	32.05	Published record	Wang et al. 2022 [6]
46	A	Guangyuan, Sichuan	106.05	32.17	Published record	Wang et al. 2022 [6]
47	A	Nanchong, Sichuan	106.05	30.80	Published record	Wang et al. 2022 [6]
48	A	Xuanhan, Sichuan	108.25	31.67	This study	This study
49	A	Taiwan Island*	121.00	23.70	Published record	Shii et al. 1997 [44]
50	A	Taiwan Island*	121.00	23.70	Published record	Chang et al. 2009 [33]
51	A	Shengzhou, Zhejiang	120.95	29.63	This study	This study
52	B	Huian, Fujian	119.00	24.94	This study	This study
53	B	Nanping, Fujian	118.13	26.68	Published record	Wang et al. 2022 [6]
54	B	Guangzhou, Guangdong	113.62	23.30	This study	This study
55	B	Shaoguan, Guangdong	114.10	25.10	Published record	Wang et al. 2022 [6]
56	B	Baise, Guangxi	105.67	24.52	Published	Wang et al. 2022 [6]

No	Cytotype	Locality	Longitude	Latitude	Source	Author
					record	
57	B	Jingxi, Guangxi	106.59	23.08	Published record	Wang et al. 2022 [6]
58	B	Hong Kong*	114.17	22.32	Published record	Kurita 1987 [16]
59	B	Quannan, Jiangxi	114.59	24.63	Published record	Shu 2010 [69]
60	B	Quannan, Jiangxi	114.58	24.78	This study	This study
61	C	Huangshan, Anhui	118.17	30.20	This study	This study
62	C	Chengkou, Chongqing	108.19	31.74	This study	This study
63	C	Conghua, Guangdong	113.92	23.80	Published record	Wang et al. 2022 [6]
64	C	Danxiashan, Guangdong	113.73	25.02	Published record	Wang et al. 2022 [6]
65	C	Shaoguan, Guangdong	113.99	24.92	Published record	Wang et al. 2022 [6]
66	C	Bama, Guangxi	107.08	24.23	This study	This study
67	C	Tianlin, Guangxi	105.70	24.58	This study	This study
68	C	Guiyang, Guizhou	106.81	26.60	Published record	Wang et al. 2022 [6]
69	C	Liupanshui, Guizhou	104.67	25.78	Published record	Wang et al. 2022 [6]
70	C	Qianxinan, Guizhou	105.36	25.67	Published record	Wang et al. 2022 [6]
71	C	Hainan*	109.70	19.20	Published record	Liu and Hsu 1989 [17]
72	C	Tongbai, Henan	113.57	32.58	This study	This study
73	C	Wufeng County, Hubei	110.93	31.46	Published record	Jiang et al. 2024 [11]
74	C	Zixing County, Hunan	113.38	25.84	Published record	Jiang et al. 2024 [11]
75	C	Jurong, Jiangsu	119.08	32.13	This study	This study
76	C	Nanjing, Jiangsu*	118.80	32.06	Published record	Cheng 2006 [68]
77	C	Nanjing, Jiangsu*	118.80	32.06	Published record	Huang et al. 2011 [19]
78	C	Yixing, Jiangsu	119.69	31.34	This study	This study
79	C	Mei County, Shaanxi	107.70	34.08	This study	This study
80	C	Mount Emei, Sichuan	103.73	29.93	This study	This study
81	C	Mount Emei, Sichuan	103.37	29.58	Published record	Shu 2010 [69]
82	C	Xuyong County, Sichuan	105.54	28.27	Published record	Wang et al. 2022 [6]
83	C	Fugong, Yunnan	98.89	27.30	Published record	Wang et al. 2022 [6]
84	C	Pingbian, Yunnan	103.69	22.93	Published record	Wang et al. 2022 [6]
85	C	Qujing, Yunnan	104.40	25.00	Published record	Wang et al. 2022 [6]
86	C	Wenshan, Yunnan	104.73	23.05	Published record	Wang et al. 2022 [6]

No	Cytotype	Locality	Longitude	Latitude	Source	Author
87	C	Xishuangbanna, Yunnan*	100.80	22.01	Published record	Liu and Hsu 1989 [17]
88	C	Yuxi, Yunnan	102.45	24.27	Published record	Wang et al. 2022 [6]

Notes: (1) The karyotypes of cytotypes A, B, and C are 2n = 14 = 8m + 6t, 2n = 15 = 7m + 8t, and 2n = 16 = 6m + 10t, respectively. (2) Geographic coordinates for localities marked with * were not explicitly reported in the cited publications. For cytogeographic visualization, approximate representative coordinates were assigned according to the named locality (e.g., city, region, or island). These coordinates are used only for plotting and should not be interpreted as exact collection-site coordinates.

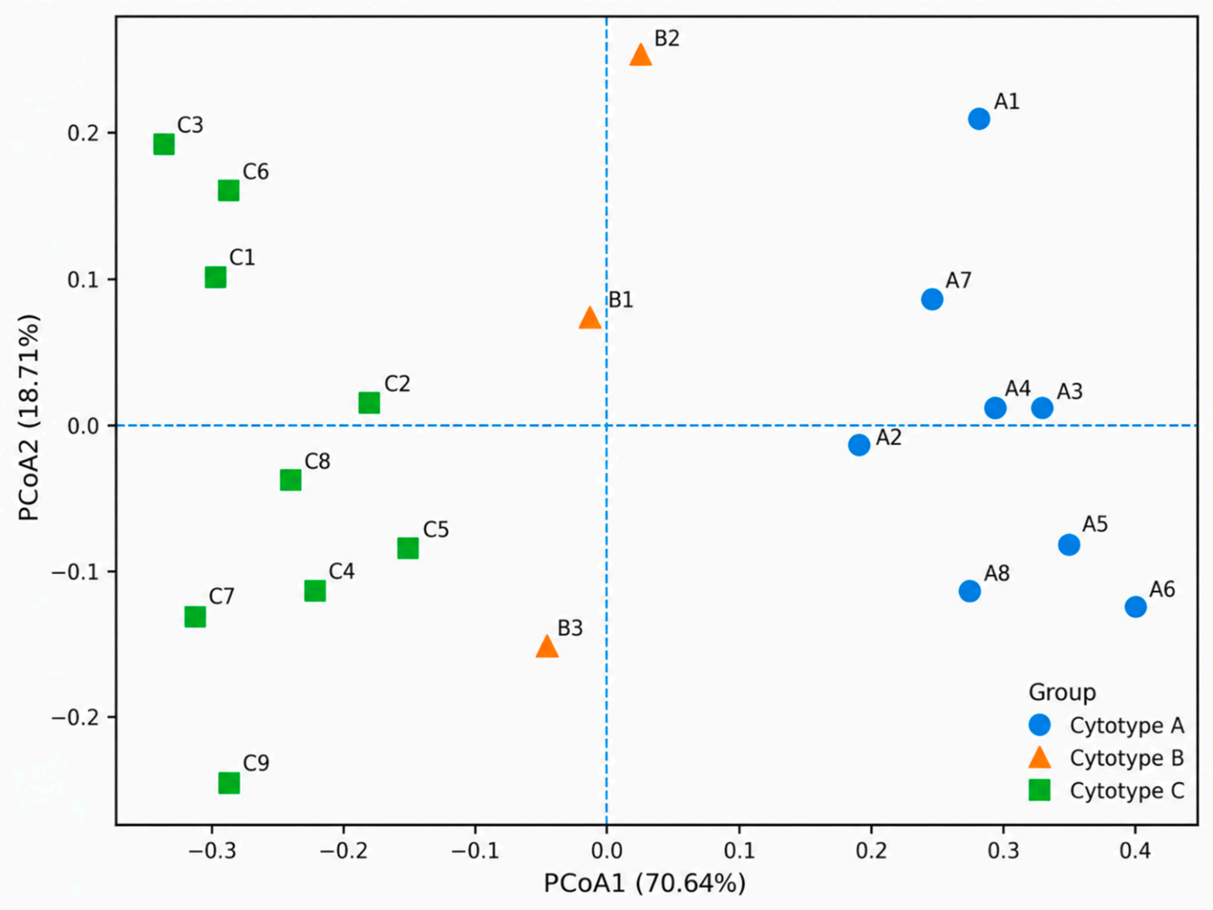

Figure S1. Principal coordinate analysis (PCoA) of 20 populations of the *Lycoris aurea* complex based on three karyotypic parameters: TDL, MCA and CVCL. Population labels correspond to those in Figure 5. The first principal coordinate (PCoA1) explains 70.64% of the total variance and captures the dominant pattern of karyotypic differentiation; the second principal coordinate (PCoA2) accounts for an additional 18.71%, reflecting secondary axes of divergence. Together, PCoA1 and PCoA2 cumulatively explain 89.35% of the total variance, indicating that the two-dimensional ordination effectively retains the major structuring signals of the original multivariate karyotypic dataset.
